# Haplotype-resolved T2T reference genomes for wild and domesticated accessions shed new insights into the domestication of jujube

**DOI:** 10.1093/hr/uhae071

**Published:** 2024-03-07

**Authors:** Kun Li, Ruihong Chen, Ayimaiti Abudoukayoumu, Qian Wei, Zhibo Ma, Zhengyang Wang, Qing Hao, Jian Huang

**Affiliations:** Key Laboratory of National Forestry and Grassland Administration on Forest Cultivation on the Loess Plateau, College of Forestry, Northwest A&F University, Yangling 712100, China; College of Horticulture, Northwest A&F University, Yangling 712100, China; Key Laboratory of National Forestry and Grassland Administration on Forest Cultivation on the Loess Plateau, College of Forestry, Northwest A&F University, Yangling 712100, China; Institute of Horticulture Crops, Xinjiang Academy of Agricultural Sciences, Urumqi 830091, China; Key Laboratory of National Forestry and Grassland Administration on Forest Cultivation on the Loess Plateau, College of Forestry, Northwest A&F University, Yangling 712100, China; Key Laboratory of National Forestry and Grassland Administration on Forest Cultivation on the Loess Plateau, College of Forestry, Northwest A&F University, Yangling 712100, China; Key Laboratory of National Forestry and Grassland Administration on Forest Cultivation on the Loess Plateau, College of Forestry, Northwest A&F University, Yangling 712100, China; Institute of Horticulture Crops, Xinjiang Academy of Agricultural Sciences, Urumqi 830091, China; Key Laboratory of National Forestry and Grassland Administration on Forest Cultivation on the Loess Plateau, College of Forestry, Northwest A&F University, Yangling 712100, China

## Abstract

Chinese jujube (*Ziziphus jujuba* Mill.) is one of the most important deciduous tree fruits in China, with substantial economic and nutritional value. Jujube was domesticated from its wild progenitor, wild jujube (*Z. jujuba* var. *spinosa*), and both have high medicinal value. Here we report the 767.81- and 759.24-Mb haplotype-resolved assemblies of a dry-eating ‘Junzao’ jujube (JZ) and a wild jujube accession (SZ), using a combination of multiple sequencing strategies. Each assembly yielded two complete haplotype-resolved genomes at the telomere-to-telomere (T2T) level, and ~81.60 and 69.07 Mb of structural variations were found between the two haplotypes within JZ and SZ, respectively. Comparative genomic analysis revealed a large inversion on each of chromosomes 3 and 4 between JZ and SZ, and numerous genes were affected by structural variations, some of which were associated with starch and sucrose metabolism. A large-scale population analysis of 672 accessions revealed that wild jujube originated from the lower reaches of the Yellow River and was initially domesticated at local sites. It spread widely and was then independently domesticated at the Shanxi–Shaanxi Gorge of the middle Yellow River. In addition, we identified some new selection signals regions on genomes, which are involved in the tissue development, pollination, and other aspects of jujube tree morphology and fertilization domestication. In conclusion, our study provides high-quality reference genomes of jujube and wild jujube and new insights into the domestication history of jujube.

## Introduction

Chinese jujube (*Ziziphus jujuba* Mill.), belonging to the Rhamnaceae, is native to China. Jujube is considered to be domesticated from wild jujube (*Z. jujuba* var. *spinosa*) [1], and both of them are the most widely cultivated and economically valuable fruit trees in the Rhamnaceae. Jujube has been widely introduced worldwide, and the jujube cultivation area has reached 2 million hectares in China [2]. Notably, the economic value and medical value of jujube and wild jujube fruits and seeds have received more attention in recent decades. Jujube and wild jujube have similar morphological characteristics but are still sharply different in important agronomic traits due to long-term selection by natural forces and human behavior, whereby wild jujube was improved from shrubby, acid-tasting, and bearing small fruit to a woody tree, sweet-tasting, and with large fruit [3, 4]. Our previous research has revealed that the jujube was domesticated from wild jujube and that the selection effect on genes determining sugar and acid content based on genome resequencing data from a small number of accessions [4]. Guo *et al*. [5, 6] elucidated that the Shanxi–Shaanxi area of China was the primary domestication center based on the genome resequencing of 493 jujube accessions, and identified candidate genes associated with several fruit traits, including size, shape, and texture, and sweetness, by an integrated genome-wide association study (GWAS). Additionally, Zhang *et al*. [3] demonstrated that some specialized metabolites, including triterpenes, in jujube fruits are believed to be directly selected during the domestication of jujube from wild jujube. However, these studies were performed based on the reference genomes assembled by short reads, and still limited jujube/wild jujube accessions were covered compared with the rich jujube germplasms, and thus the genetic basis of the domestication of jujube remains largely unexplored. Therefore, the complete and accurate reference genome of jujube and its wild relative will lay a solid foundation for understanding the genome evolution of jujube and identifying the functional genes linked to these important traits.

Benefiting from the advances in long-read sequencing technologies, it is possible to obtain a gap-free genome that will open avenues for exploring unique genetic and structural variations (SVs) in the ‘genomic dark matter’ regions, such as telomeres, transposable elements (TEs), and segmental duplications [7]. For highly heterozygous diploid and polyploid organisms, a haplotype-phased genome represents the ultimate goal of dissecting genomes and allows breeders to capture the effect of allelic gene function [8]. In recent years, based on the integration of multiple sequencing techniques and assembly strategies, complete haplotype-resolved telomere-to-telomere (T2T) genomes have been obtained in some horticulture plants, such as kiwifruit [9, 10], cassava [11], lemon [12], and melon [13]. So far four versions of the genome assembly of jujube, including *Z. jujuba* ‘Dongzao’ [14, 15] (DZV1, DZV2), *Z. jujuba* ‘Junzao’ [4] (JZV1) and *Z. jujuba* var. *spinosa* ‘Suanzao’ [16] (SJV1), has been achieved. These reported draft jujube genomes still contain a large number of unclosed gaps and fragmented unanchored sequences [4, 14, 16]. Recently, Yang *et al*. [15] generated a new version of the gap-free T2T genome assembly of ‘Dongzao’ (DZV2) using long-read sequences. However, the mosaic assemblies likely contain unassembled or unplaced allelic variations that underlie crucial selected traits. To fully understand the genomic variation of jujube flora, a high-quality haplotype-resolved genome will greatly promote the understanding of the complex traits of jujube plants.

In this study, a dominant dry-eating jujube cultivar, ‘Junzao’ (JZ), and a wild sour jujube accession (SZ) from Qingjian county, China, was chosen for T2T and gap-free genome assembly using PacBio HiFi and ONT as well as a variety of assembly strategies. The haplotype-phased genomes for both JZ and SZ were assembled at T2T levels and genome-wide variants were further identified. In addition, the population structure and domestication events were analyzed based on large-scale of resequencing data from 672 accessions. We hope to provide high-quality reference T2T genome sequences for jujube and wild jujube and new insights into jujube domestication.

## Results

### Genome sequencing and assembly

**Figure 1:**
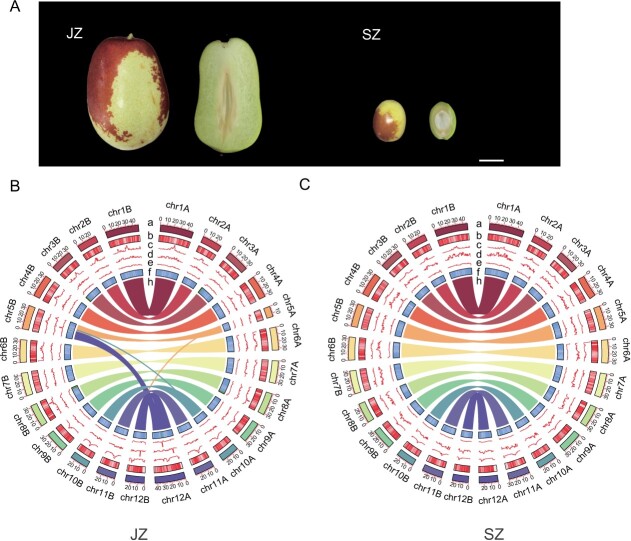
Assembly and genomic features of *Z. jujuba* Mill. ‘Junzao’ (JZ) and a wild jujube accession (SZ). **A** Fruits of JZ and SZ. **B**, **C** Circos plot of JZ and SZ haplotype-resolved gap-free genomic features. (a) Chromosome length; (b) gene density along each chromosome (red, number of genes per 100 kb); (c) LTR/Gypsy element content; (d) LTR/Copia content; (e) repeat content along each chromosome; (f) GC content (nucleotides per megabase); (h) the innermost part of the plot represents the collinear relationship between the haplotype-resolved genomes. Using 1-Mb intervals, the TE density, Copia density, and Gypsy density were determined in the JZ and SZ genomes.

To perform gap-free and haplotype-resolved assembly of JZ and SZ genomes, we generated 39.53 Gb (~94.99× depth) of clean Illumina paired-end reads for JZ and 43.15 Gb (~101.98× depth) for SZ (Fig. 1A, Supplementary Data Table S1). Based on *k*-mer analysis (*k* = 19), we estimated that the genome size of JZ was ~416.14 Mb, with a heterozygosity rate of 0.91% and ~40.74% repetitive sequences throughout the genome (Supplementary Data Fig. S1). Similarly, the estimated genome size of SZ was around 423.11 Mb, with a heterozygosity rate of 0.92% and ~40.65% repetitive sequences. Furthermore, utilizing the ONT sequencing platform, we acquired 32.51 Gb pass bases for JZ and 33.39 Gb for SZ (Supplementary Data Table S2). Using the PacBio HiFi platform, we obtained HiFi CCS reads of 32.39 Gb in JZ and 60.79 Gb in SZ after filtration.

The preliminary assembly for JZ was 399.25 Mb containing 65 contigs, while the preliminary assembly of SZ was 391.18 Mb with 48 contigs (Supplementary Data Table S3). The contig N50 lengths for JZ and SZ were ~14 times longer than those of the earlier JZV1 and SJV1 genomes (Table 1). Following the elimination of bacterial and organelle contaminants from the primary assembly, the 52.36 Gb Hi-C data of JZ and 45.37 Gb Hi-C data of SZ were aligned to 65 and 48 contigs, respectively, utilizing 3D-DNA software (v180419) (Supplementary Data Fig. S2) [17]. After Hi-C assembly and manual adjustments, JZ exhibited 385.71 Mb of genome sequence, which were anchored to 12 chromosomes with one gap and 47 unanchored contigs (Supplementary Data Fig. S3, Supplementary Data Tables S4 and S5). Similarly, SZ exhibited 397.79 Mb anchored to 12 chromosomes, with 33 gaps and 12 unanchored contigs. The unanchored contigs predominantly originated from the rDNA sequences of chloroplasts and mitochondria, along with the genomic material of cellular organelles, encompassing redundant sequences. After filling gaps and checking the coverage of gap regions, chromosome ID and orientation were adjusted according to JZV1 (LPXJ00000000.2) (Supplementary Data Fig. S4). Finally, two gap-free reference genomes were generated, the final assembly of JZ had a size of 385.80 Mb with a contig N50 value of 31.07 Mb, and the final assembly of SZ was 375.15 Mb with a contig N50 value of 30.84 Mb (Supplementary Data Fig. S5, Supplementary Data Table S6).

**Table 1:** Comparison of jujube and wild jujube genome assembly with previously published genomes.

	‘Junzao’ (JZ) (GCA_037113555.1)	Wild jujube (SZ) (GCA_037113545.1)	‘Dongzao’ DZV2(GWHCBFT 00000000.1)	‘Junzao’ (JZV1) (LPXJ00000000.2)	SJV1 (JAEACU0000 00000.1)
Size of assembly (bp)	385 801 156	375 153 944	393 332 932	362 583 438	405 636 987
Contig N50 (bp)	31 079 923	30 843 711	32 986 920	2 172 811	2 216 458
Number of contigs	12	12	12	269	907
BUSCOs of genome (%)	98.70	98.20	98.50	96.90	97.70
Number of gaps	0	0	0	222	381
Centromeres	12	12	12	\	\
Telomeres	24	24	24	\	\
Protein-coding genes	27 651	26 658	29 633	28 223	31 115
BUSCO genes (%)	96.9	96.6	93.70	91.50	93.10
Annotation proteins	26 634	25 703	29 633	26 222	28 863
Transposable elements (Mb)	212.21	203.61	220.88	193.52	226.65
TE ratio in genome (%)	55	54.27	56.16	53.37	55.88

According to the results of *k*-mer analysis, JZ and SZ have heterozygosity levels of 0.91 and 0.92%, respectively. We conducted haplotype-resolved genome assemblies of JZ and SZ, which were obtained based on HiFi reads and assisted by Hi-C and ONT ultra-long reads. We successfully anchored the 29 contigs of JZ onto 24 pseudochromosomes, and the 28 contigs of SZ onto 24 pseudochromosomes (Supplementary Data Fig. S6, Supplementary Data Table S7). All gaps on the 24 pseudomolecules of JZ and SZ haplotype genomes were filled, and telomeres at both ends of all chromosomes were identified. These two haplotype combinations had lengths of ~767.81 Mb for JZ and ~794.58 Mb for SZ, designated as JZHapA (383.00 Mb) and JZHapB (384.80 Mb) for JZ, and SZHapA (385.83 Mb) and SZHapB (373.42 Mb) for SZ (Fig. 1B and C, Supplementary Data Table S6).

We found a chromosome fusion or fission event involving chromosome 5 and chromosome 12 in JZHapA (Fig. 1B, Supplementary Data Fig. S7). IGV software was used to examine the coverage of HiFi and ONT reads at the end of the syntenic region of chromosome 12, and the sequences in the interval were covered by reads (Supplementary Data Fig. S8). It was observed that there were significant collinearity regions in JZHapA chromosome 12 and JZhapB chromosome 5. We extracted chromosomes 5 and 12 of JZhapA and JZhapB to further examine the continuous interaction signals on the Hi-C heat map and confirmed that these signals were not misassembled (Supplementary Data Fig. S9).

We predicted 29 491 and 29 639 coding genes in JZHapA and JZHapB, which were 1840 and 1988 more than chimera genomes, respectively (Supplementary Data Table S8). Meanwhile, we predicted 28 245 and 27 810 coding genes in the SZHapA and SZHapB genomes, 1587 and 1152 more genes than in the chimera genomes. Functional annotation of genes was performed, and the results showed that 94.44–94.84% of genes were annotated in the four haplotype-resolved genomes (Supplementary Data Table S9). Repetitive sequences were identified in the two haplotype genomes of JZ, with a total of 202.43 and 204.78 Mb of repetitive sequences identified, accounting for 52.86% of HapA and 53.22% of HapB, respectively (Supplementary Data Fig. S10A and B, Supplementary Data Table S10). In the two haplotype genomes of SZ, identified repetitive sequences amounted to 209.57 and 198.51 Mb, accounting for 54.32 and 53.16% of HapA and HapB, respectively. Among all classifications of TEs, long terminal repeat (LTR) elements were predominant, accounting for ~29.99 and 30.27% in JZHapA and JZHapB, and 29.91 and 29.19% in SZHapA and SZHapB genomes (Supplementary Data Fig. S10C and D).

### Quality assessment of genome assemblies

Genome integrity was evaluated as a high integrity level of 98.7% for JZ and 98.2% for SZ using Benchmarking Universal Single-Copy Orthologs (BUSCO). Additionally, the assessment of haplotype genomes demonstrated that JZHapA and JZHapB achieved genome completeness with BUSCOs at 99 and 98.9%, while SZHapA and SZHapB exhibited haploid genome completeness with BUSCOs at 99 and 98.9% (Supplementary Data Table S11). The Merqury evaluation of the two haplotype assemblies of JZ and SZ showed high accuracy, with QV values in the range of 53.31–56.03. We further examined the genome assembly according to the standards recommended by the Earth BioGenome Project (EBP) (https://www.earthbiogenome.org/assemblystandards). According to the standards, both JZ and SZ assemblies have achieved all quality categories except base accuracy (Supplementary Data Table S12). The Merqury evaluation shows that the QV value of JZ meets the 7.C.Q50 standard at 50.17, with *k*-mer completeness at 83.54, satisfying the 4.5.Q30 standard. In the case of SZ, the QV is 42.76, meeting the 6.7.Q40 standard, and *k*-mer completeness is at 84.11, meeting the 4.5.Q30 standard.

### Identification of telomeres and centromeres

In total, we identified the telomeres in each chromosome of both chimeric and haplotype-resolved JZ and SZ genomes successfully (Supplementary Data Table S6). The number of telomere repeats in the chimeric genomes of JZ and SZ were in the range of 835–2807 and 1039–2794, with a mean value of 2219 and 2020, respectively. The telomere repeat sequences for both JZHapA and JZHapB were identified on 24 chromosomes, with repeat numbers ranging from 1501 to 2776, while the repeat numbers of SZHapA and SZHapB were between 1004 and 2717.

The centromere candidate regions were determined in each chromosome of JZ and SZ, and significant differences in tandem repeat coverage, centromere length, and position between chromosomes were observed (Supplementary Data Table S6). The centromere boundaries in the two haplotype genomes of JZ ranged from 0.11 to 1.82 Mb, while the centromere boundaries in the two haplotype genomes of SZ ranged from 0.12 to 3.27 Mb. The tandem repeat coverage ratio in JZ and SZ spanned from 7.76 to 77.94%. Additionally, the Hi-C interaction heat maps also indicate fewer interactions detected in these regions, suggesting that these regions with highly tandem-repeated sequences are centromeres (Supplementary Data Figs S11 and S12).

There were 138 and 190 protein-coding genes located within the centromeres of JZHapA and JZHapB, and 180 and 178 protein-coding genes located within the centromeres of SZHapA and SZHapB, respectively. Through Gene Ontology (GO) functional annotation, these genes of the JZHapA and JZHapB were significantly enriched in multiple cellular components, including ‘DNA biosynthetic process’, ‘spindle pole’, ‘microtubule organizing center’, and ‘microtubule nucleation’, and these genes of the SZHapA and SZHapB were significantly enriched in ‘cell differentiation’, ‘elongator holoenzyme complex’, ‘microtubule motor activity’, and ‘kinetochore’ (Supplementary Data Tables S13 and S14).

### Comparative genomics analysis between jujube and wild jujube

**Figure 2:**
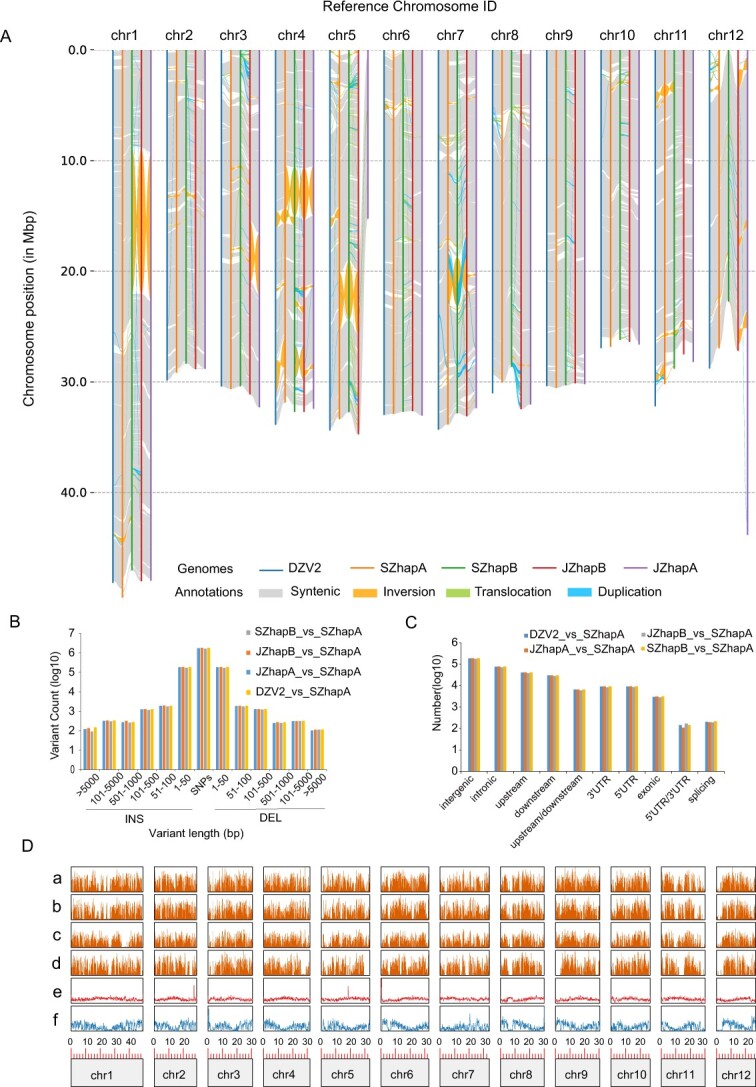
Comparative genomics analysis of jujube and wild jujube genomes. **A** Syntenic analysis of JZHapA, JZHapB, SZHapA, SZHapB, and DZV2 genomes. **B** Distribution of variation length was compared using the SZHapA genome as reference and ordinate numbers were logarithmically transformed. **C** Annotation of indel (<50 bp) variation for comparison of genomic variation. UTR3, 3′ untranslated region; UTR5, 5′ untranslated area; upstream/downstream, upstream or downstream 1-kb intronic region; exonic, exonic region; intergenic, intergenic region; splicing, within 2 bp of the splicing site. **D** Distribution of indel (>50 bp) variants on chromosomes: (a) DZV2 versus SZHapA; (b) JZHapA versus SZHapA; (c) JZHapB versus SZHapA; (d) SZHapB versus SZHapA; (e) repeat sequence content; (f) gene density (indels use a 1-Mb window, repeats and gene densities use a 100-kb window).

We aligned JZHapA, JZHapB, SZHapA, SZHapB, and the newly published DZV2 genomes to each other. A comparison between the two haplotypes, HapA and HapB, of JZ showed 1 543 136 single-nucleotide polymorphisms (SNPs), 162 542 insertions, 163 198 deletions, 46 inversions, 198 translocations, and 198 duplications (Supplementary Data Table S15). Between the two haplotypes, HapA and HapB, of SZ, there were 1 616 437 SNPs, 173 203 insertions, 172 488 deletions, 60 inversions, 1156 translocations, and 133 duplications. In addition, ~81.60 and 69.07 Mb of SVs were found between the two haplotypes within JZ and SZ, accounting for 21.51 and 18.49% of the genome size, respectively. Syntenic analysis revealed large-scale SVs on chromosomes 1, 3, 4, and 12 between JZHapA and JZHapB, and on chromosomes 4, 5, and 7 between SZHapA and SZHapB (Fig. 2D, Supplementary Data Fig. S7).

Then we aligned JZHapA, JZHapB, DZV2, and SZHapB genomes with SZHapA. There were 3 240 381–5 193 414 SNPs and 345 651–375 916 insertions/deletions identified in the pairwise alignments of assemblies (Supplementary Data Table S15). In several comparison groups, the length of insertions/deletions was mainly within 50 bp, and most of the mutation sites were concentrated in intergenic regions (Fig. 2B, Supplementary Data Table S16). Among several groups of exon position variations, DZV2 showed the most. The distribution of indel/absence >50 bp was similar in chromosome distribution among JZHapA, JZHapB, and DZV2 (Fig. 2D). A total of 70 inversions, 176 translocations, and 227 syntenic regions covering >343.23 Mb were identified between DZV2 and SZHapA. The results showed that there are two major inversions between DZV2 and SZhapA on chromosomes 4 and 11. Compared with the SZHapA genome, 64 inversions and 166 translocations as well as 200 syntenic regions covering >315.29 Mb were detected in the JZHapA genome, and 74 inversions and 203 translocations as well as 257 syntenic regions covering >325.91 Mb were detected in the JZHapB genome. Large-scale SVs were also found between SZHapB and JZHapB on chromosomes 4, 5, and 7. Subsequent examination of the raw read coverage at these SV sites confirmed the authenticity of these variants, further confirming the correctness of the JZ and SZ haplotype genome assembly (Supplementary Data Figs S13 and S14). We pooled several groups of genes that were affected by SVs in the comparison, and GO enrichment analysis indicated that there were gene associations with polysaccharide binding in molecular functions and lipid catabolic process in biological process’ (Supplementary Data Table S17). KEGG pathway analysis indicated associations with phenylpropanoid biosynthesis, starch and sucrose metabolism, glycerophospholipid metabolism, and flavonoid biosynthesis (Supplementary Data Table S18).

### Allele-specific expression patterns between haplotypes

To assess differentially expressed alleles (DEAs), 22 574 and 23 042 alleles were identified in the JZ and SZ haplotype genomes, respectively. The RNA-seq reads of jujube fruit at three mature stages (expanding fruit, semi-red fruit, and full-red fruit) were compared, and the overall comparison rate was >99.69% (Supplementary Data Table S19). The expression patterns of a large number of allelic pairs differed between haplotypes and at different periods (Supplementary Data Fig. S15). Then, DEAs in different periods were analyzed (|log_2_[fold change]| ≥ 1). A total of 4931 and 2720 alleles of JZ and SZ were differentially expressed at different periods, respectively, while the rest were equivalently expressed alleles (EEAs). To evaluate the natural selection of alleles, *K*_a_ and *K*_s_ values were calculated for all allelic pairs of JZ and SZ. The *K*_a_ and *K*_s_ values of 88.01% of DEAs and 89.89% of EEAs in JZ were <0.05, while those of 89.81% of DEAs (Supplementary Data Table S20) and 89.66% of EEAs (Supplementary Data Table S21) in SZ were <0.05. These results indicated that the sequence consistency of DEAs and EEAs was high. However, DEAs of JZ and SZ exhibited higher *K*_a_/*K*_s_ values than those of EEAs (*P* = 0.27and 0.46), and *K*_s_ values (*P* = 2e−137 and 2e−16) were higher than those of EEAs (Supplementary Data Fig. S16). About 16.3% (445 out of 2720 alleles) of JZ were clearly selected for purification (*K*_a_/*K*_s_ < 0.1), while 4.89% (133 out of 2720 allelic pairs) of genes showed possible positive selection (*K*_a_/*K*_s_ > 1). About 17.1% (845 out of 4931 alleles) of SZ underwent clear purification selection (*K*_a_/*K*_s_ < 0.1), while 4.28% (211 of 4931 allelic pairs) of genes showed possible positive selection (*K*_a_/*K*_s_ > 1). We further studied the DEAs of JZ and SZ. DEAs in JZ are mainly concentrated in the biosynthesis of amino acids and ubiquitin-mediated proteolysis, while many DEAs in SZ are involved in glycolysis/gluconeogenesis, biosynthesis of nucleotide sugars, α-linolenic acid metabolism, and pyruvate metabolism (Supplementary Data Fig. S17).

### Population structure and evolution of jujube

**Figure 3:**
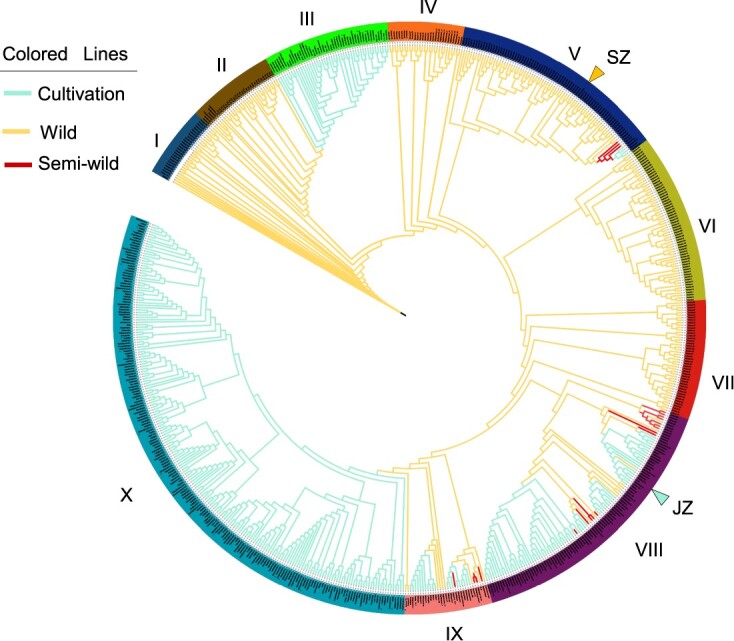
Phylogenetic analysis of jujube and wild jujube population. NJ phylogenetic tree of jujube and wild jujube accessions inferred from whole-genome SNPs. *Ziziphus spina-christi* was used as an outgroup. The yellow triangle represents the location of SZ, and the cyan triangle represents the location of JZ. The yellow line of the phylogenetic tree represents the wild accession, the cyan line indicates cultivated populations, and the red line indicates semi-wild populations. Different colors on the outer circle indicate different accessions, divided into 10 subgroups.

To further elucidate the population structure and genetic relationships between wild jujube and cultivated jujube, resequencing data from 672 individuals were subjected to population analysis. This diverse dataset included 359 cultivated, 291 wild, and 22 semi-wild jujube samples, offering comprehensive insights into domestication and population structure in wild jujuba and jujube (Supplementary Data Table S22). The dataset, aligned to the JZ genome, uncovered 30 517 287 high-quality SNPs, averaging 79.09 SNPs per kilobase. Further filtering yielded a basic set of 33 700 SNPs, which was selected to construct a neighbor-joining (NJ) tree with *Z. spina-christi* (Christ’s thorn jujube) as the outgroup (Fig. 3, Supplementary Data Fig. S18). In general, phylogenetic analysis delineated distinct clades, notably identifying clade I as the ancestral group, primarily composed of wild jujubes from Shandong province. Supporting this, a *Z. miojujuba* leaf fossil (12–14 Mya) which closely matches the leaf morphology of wild jujube, were discovered in Linju county of Shandong [18, 19]. Clade II and clade IV derived from the common ancestral clade I, and were further subdivided into cultivated and wild jujubes or semi-wild jujubes, respectively, leading to two domestication events of jujube. Comparison among clades showed that the *F*_ST_ value between clade III and other clades was higher, indicating that clade III and other clades had a greater degree of differentiation (Supplementary Data Fig. S19A). Phylogenetic clades exhibited pronounced geographic distribution patterns (Supplementary Data Fig. S19B). Wild jujubes and jujubes in clade II and clade III were mainly native to the Shandong Province and Hebei Province near the lower reach of the Yellow River. Clades IV and V were mostly composed of germplasm from Hebei province. These results indicated that cultivated jujube originated from a common ancestor, but cultivated jujube was domesticated at two independent domestication centers, i.e. Shandong province near the lower reaches of the Yellow River, and the region of Shanxi–Shaanxi Gorge at the middle Yellow River.

**Figure 4:**
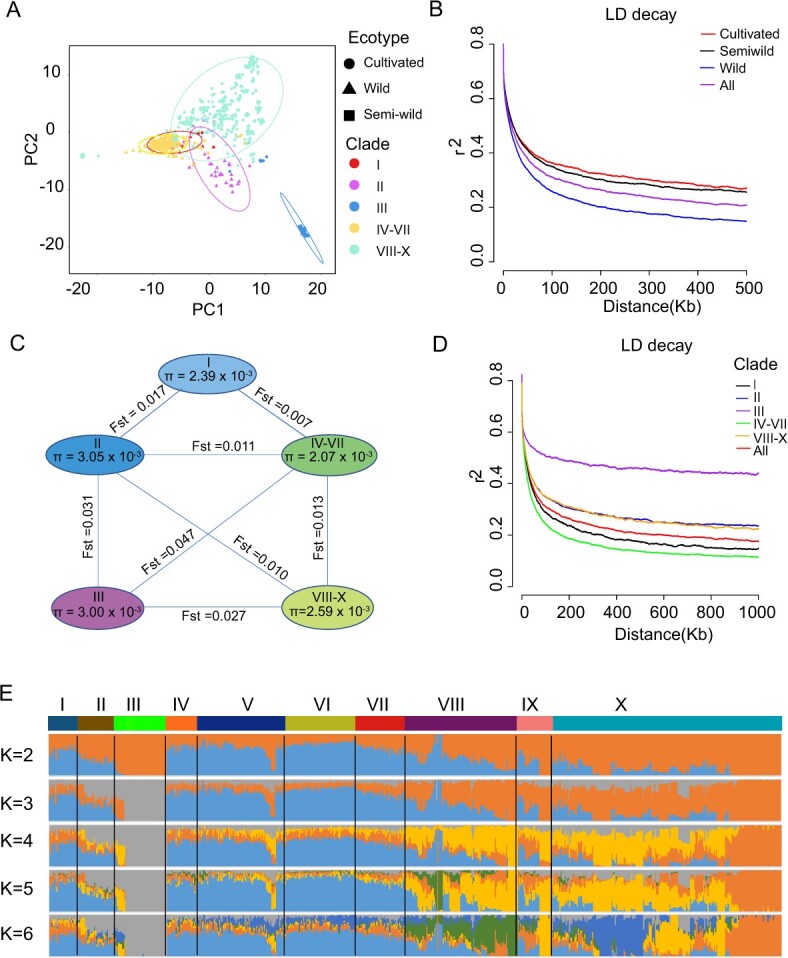
Population structure of jujube and wild jujube accessions. **A** PCA plot of 359 cultivated jujube, 291 wild jujube, and 22 semi-wild jujube accessions. Wild jujube accessions are indicated by triangles, cultivated jujube accessions by circles, and semi-­wild populations by boxes, different colors describe different clades in the NJ tree. **B** Decay of LD in all samples, cultivated jujube, wild jujube, and semi-wild jujube. **C** Nucleotide diversity (π) and population differentiation (*F*_ST_) in various branches of the NJ tree. **D** Attenuation of LD of jujube in NJ tree branches. **E** Population structure of major jujube categories estimated by ADMIXTURE. Each color represents an ancestral population. Each addition is represented by a bar, and the length of the colored segment in each bar indicates the proportion contributed by that ancestral population.

Principal component analysis (PCA) showed distinct clustering patterns among wild, cultivated, and semi-wild jujube accessions. Wild jujube accessions formed a close cluster, while cultivated jujube accessions exhibited a more scattered clustering pattern and overlapped with the cluster formed by wild jujubes, and semi-wild accessions were distributed between cultivated accessions and wild accessions (Fig. 4A). This indicates that jujube underwent genetic diversification during domestication, resulting in a wider range of genetic variation of the jujube population. Genome-wide decay of linkage disequilibrium (LD) indicated that wild jujube exhibits a faster LD decay rate compared with cultivated and semi-wild jujube (Fig. 4B). In contrast, LD decay in cultivated jujube was found to be the slowest among the three groups. At the same time, we observed that clade II and clade III were significantly separated from other clades on PCA, and *F*_ST_ and nucleotide diversity showed that clade II and clade III had higher genetic diversity and greater genetic differences compared with other populations (Fig. 4C and D).

To further investigate the population structure of the wild jujube and jujube population, we ran ADMIXTURE software to analyze *K* values from 2 to 6 (Fig. 4E, Supplementary Data Fig. S20). ADMIXTURE clustering analysis revealed a complex population structure of the cultivated jujube population while population structure was simple in wild jujube. When *K* = 2, cultivated jujube and wild jujube were clearly divided. When *K* = 3, cultivated jujube in clade III had clearly differentiated from clade II and displayed distinct genetic composition. When *K* = 5, the differentiation between these cultivated accessions was evident in the population mix map. When *K* increased from 3 to 6, wild jujube had less genetic admixture and maintained purer ancestries. The internal population structure of cultivated jujube (clades VIII–X) showed admixed genetic structures, and the clade III genetic background maintained pure or nearly pure ancestry. These results indicate that cultivated jujube accessions have indeed experienced two independent domestication events, and only diversified through one route. On the whole, the population structure clearly described the geographical origins of jujube. Geographic clustering patterns of the phylogenetic tree and population structure shed light on the introduction and development processes during domestication (Supplementary Data Fig. S21).

### Adaptive evolution of jujube

To elucidate potential regions and genes undergoing selection during the improvement and domestication of jujube, we conducted an extensive sampling of genotypes. Utilizing a 100-kb sliding window, we calculated the reduction of diversity (ROD) and pairwise population fixation index (*F*_ST_) between wild and cultivated jujube populations. Selective sweep regions (window step 10 kb) with the top 5% of *F*_ST_ (*F*_ST_ > 0.041) and ROD (ROD >0.569) values were designated as highly divergent candidate regions (Supplementary Data Fig. S22A). A total of 389 candidate selective sweep regions exhibited increased differentiation between wild and cultivated jujube, with 300 genes identified in these selective sweeps (Supplementary Data Table S23). These 300 candidate genes showed that GO terms involved in transporter activity, pollination, phloem or xylem histogenesis, tissue development, photosynthesis, and multi-multicellular organism process were significantly enriched (Supplementary Data Table S24). In particular, several functional genes associated with jujube fruit quality and stress resistance were selected in these regions, such as *Sugartransporter* (*ST*) [20], *Pectin acetylesterase *(*PAE*) [21], *UDP-Glycosyltransferase *(*UGT*), *G-type lectin S-receptor-like serine* (*SRK*) [22], and *leucine rich repeat *(*LRR*) [23] (Supplementary Data Fig. S22B and C). We identified a RING-finger protein (BIG BROTHER) as a repressor of plant organ growth in the selective sweeps, which may have been selected during the domestication of wild jujube to jujube [24].

## Discussion

Assembling a genome that is both highly heterozygous and rich in repetitive sequences is still a substantial challenge. With the advance of long-read sequencing technology, it is possible to assemble the genome at T2T level and the diploidy could also be resolved. Haplotype­-based genetic variants directly reflect essential changes along single chromosomes and the haplotype genome has an essential value in genome structure, evolution, linkage analysis, association studies, allele analysis, and population genetics. Since the first genome of Chinese jujube was published in 2014 by Liu *et al*. [14], four genome versions of jujube have been published and updated, which constitute valuable genomic resources for the study of Chinese jujube [4, 15, 16]. However, the mosaic assemblies are likely to have overlooked structure and allelic variations underlying important selected traits. In this study we successfully assembled two haplotype-resolved and gap-free genomes of a dominant jujube cultivar, ‘Junzao’ (JZ), and the wild jujube SZ employing a combined approach, with JZHapA and JZHapB sizes of 383.00 and 384.80 Mb for JZ and SZHapA and SZHapB sizes of 385.83 and 373.42 Mb for SZ, respectively (Supplementary Data Table S6). Telomeres, centromeres, and free gaps in all assemblies of JZ and SZ suggest that each of the 12 chromosomes in both genomes possesses a comprehensive T2T structure. Notably, these assemblies represent a substantial improvement compared with previous genomes in terms of completeness and accuracy [4, 14, 16]. Recently, another dominant jujube cultivar, ‘Dongzao’, was sequenced at T2T level with high quality [16]; however, this assembly was not haplotype-resolved. As reflected by the two assemblies generated in our study, i.e. chimeric and haplotype-resolved assemblies, there were a few differences in genome size, gene numbers, genome structure, etc. Thus, the chimeric assembly would ignore the diploid nature to some extent. In this study, the haplotype-resolved T2T genomes for both cultivated jujube and wild jujube provide essential references for dissecting how the complex traits of jujube associate with the genome.

During jujube domestication an imprint may have been left on its genome [25]. Continuous selection and breeding efforts have significantly altered the sugar content of modern fruits [26]. However, there is a dearth of comprehensive comparative genomic analyses between jujube and its wild counterpart. In this study genome-wide variation analyses were performed on three jujube T2T genomes (JZ, SZ, and DZV2) with SZHapA as a reference, and three types of variations (SNP, indel, and SV) were characterized (Fig. 2). Syntenic analysis revealed large-scale inversions on chromosomes 1, 3, 4, and 12 between JZ’s two haplotypes, JZHapA and JZHapB. In addition, large-scale inversions were found between the two SZ haplotypes, SZHapA and SZHapB, on chromosomes 4, 5, and 7. Heterozygous haplotypes exhibited prevalent SNPs, indels, and SVs, indicating extensive genetic diversity. A large number of structural variations were also detected between the genomes of JZ and DZV2, with similarities in indel distributions. Large numbers of structural variations were also detected between the genomes of JZ and DZV2, with similarities in indel distributions [4, 5]. These findings highlight SV-affected genes as promising candidates for further research on the genetic basis of jujube fruit sweetness. In previous reports, genes related to sugar/acid metabolism, fruit size, and some specific metabolites were subject to domestic selection [4, 6]. Our selection analysis based on more accessions revealed some new signals on genomes, such as tissue developments and pollination, and particularly a BIG BROTHER gene controlling organ size was also selected. This might reflect the selection effect of human behavior improving the wild jujube shrub to woody jujube plants.

It is generally known that jujube originates from wild jujube, with some semi-wild jujubes acting as intermediaries, allowing a distinct separation between cultivated and wild jujube populations [5, 6]. In the present study, 672 accessions covering >50% of known jujube cultivars or populations were subjected to phylogenetic and population analysis. Notably, the results showed that two interdependent jujube groups were derived from wild counterparts, affirming the hypothesis of ‘multiple ways of jujube domestication from wild jujube’ proposed by Qu and Wang [27] and Liu and Wang [19]. Jujube was initially domesticated from local sites at the lower reach of the Yellow River. Meanwhile, wild jujube was diffused to the middle reach of the Yellow River (Shanxi–Shaanxi Gorge) and was domesticated to diverse jujube cultivars, which is consistent with the report by Guo *et al*. [5]. Compared with the domestication of cultivated jujube in the Shanxi–Shaanxi Gorge, the origin of wild jujube was suggested to be in the lower reach of the Yellow River in Shandong province. This discovery was in accordance with the archaeological discovery in Linqu County, Shandong province, of a fossil *Z. miojujuba* leaf with a very similar morphology to that of wild jujube [28]. At the same time, the presence of varied germplasm ecotypes within the evolutionary branches of wild and cultivated jujube raises questions about potential misclassifications based on morphology [29].

Overall, using long-read sequencing, we successfully generated a gap-free T2T haplotype-resolved genome assembly of cultivated jujube JZ and wild jujube SZ. The availability of these high-quality genome assemblies serves as a valuable resource for future research endeavors in the field of jujube genomics. It opens up new possibilities for refining breeding strategies, enhancing crop productivity, and unraveling the genetic basis of traits in this economically important fruit tree species.

## Materials and methods

### Plant sampling and DNA extraction

Fresh leaflets of jujube cultivar ‘Junzao’ (JZ) and wild jujube (SZ) for genome sequencing were collected at the Jujube Experimental Station of Northwest A&F University (Qingjian, Shaanxi Province, China, 37.13° N, 110.09° E), and stored in liquid nitrogen until DNA extraction and sequencing.

### Library preparation and DNA sequencing

High-quality genomic DNA of JZ and SZ was extracted by a modified CTAB method [30], and the quality of genomic DNA was determined. For PacBio HiFi sequencing, libraries were prepared using Pacific Biosciences SMRTbell Express Template Prep Kit 2.0 (Pacific Biosciences, USA) according to the standard protocol. An SMRT cell sequencing library was constructed and then sequenced on a PacBio Sequel II system (Pacific Biosciences, CA, USA).

For Oxford Nanopore Ultra-long sequencing, the libraries were prepared using the Oxford Nanopore SQK-LSK109 kit according to the standard protocol. The purified library was loaded onto primed R9.4.1 Spot-On Flow Cells and sequenced using a PromethION sequencer (Oxford Nanopore Technologies, Oxford, UK).

For Illumina sequencing, qualified DNA samples were randomly interrupted to prepare a 350-bp inserted fragment library. Then, the Agilent 2100 Bioanalyzer system was used to verify the insertion size of the library, and qPCR was performed to ensure the effective quantitative concentration of the library. The library was sequenced on the Illumina NovaSeq 6000 platform (Illumina, San Diego, CA, USA). The resulting short reads were used for genome survey analysis.

Finally, the young leaf cells of JZ and SZ were immobilized with paraformaldehyde, a Hi-C library was established and the Illumina NovaSeq 6000 platform was used for sequencing.

### Plant material, RNA library construction, and sequencing

Total RNA was extracted from young leaves, flowers, young stems, and fruits of JZ and SZ. Sequencing libraries were generated using the NEBNext^®^ Ultra™ RNA Library Prep Kit for Illumina^®^ (#E7530L, NEB, USA) following the manufacturer’s recommendations. Then, the libraries were subjected to 150-bp paired-end sequencing on an Illumina platform. For Oxford Nanopore cDNA sequencing, RNA samples were pooled to construct a cDNA library and sequenced on a PromethION sequencer (Oxford Nanopore Technologies, Oxford, UK).

### Genome assembly and quality assessment

A high-quality T2T genome was constructed by means of gap filling, telomere extension, and error correction using a hybrid assembly method using ONT ultra-long data and HiFi data. The working process was as follows. Primarily, for the PacBio raw subreads, consensus reads (HiFi reads) were generated using CCS software (v6.0.0; parameters: -min-passes 3 -min-snr 2.5 -top-passes 60; https://github.com/PacificBiosciences/ccs). For ONT ultra-long raw data, Filtlong (v0.2.4; https://github.com/rrwick/Filtlong) was used to filter fragments smaller than 10 kb. We used Porechop (v0.2.4; https://github.com/rrwick/Porechop) to filter the joint sequence and reads with length >=30 kb and mean read quality scores >90% were retained for assembly using Filtlong software.

Subsequently, the ONT ultra-long pass reads and the PacBio HiFi clean reads were used for mosaic assembly. In short, we used the software Hifiasm (v0.16.1) for preliminary assembly of HIFI reads. The ONT ultra-long reads were aligned to the preliminary assembled genome to identify gaps and further fill the gaps. For genome haplotype assembly, the hifi+ont + hic assembly mode of Hifiasm software (v0.18.2-r467; parameters: Hifiasm-0.19.5-r587/hifiasm-0.19.5/hifiasm -f 0 -l 2 --ul ont_ul.fq.gz --h1 hic_R1.fq.gz --h2 hic_R2.fq.gz ccs.fa) was used to conduct preliminary assembly of JZ and SZ. Assembly contamination, including mitochondrial, chloroplast genome contamination, and bacterial contamination, was removed with Minimap2 (2.17-r941) [31]. To further optimize the assembly results, ALLHiC (v0.9.8) and 3D-DNA (v180419) were used to divide contigs into different clusters according to the closeness of association between different contigs in the valid data [17, 32]. Contigs within the chromosome group were evaluated, oriented, and removing redundancies to obtain a chromosome-level genome sequence. The gaps in the genome were closed by Winnowmap (v1.11, k = 15, -- MD) [33] with HiFi reads and corrected ONT long reads. If the aligned region perfectly spanned both ends of the gap, we selected the longest and best alignment region to replace and complete the gap filling. HiFi reads (≥10 kb) were again used to compare with gap-filled genomes for error correction (k = 15 greater-than distinct = 0.9998 — MD-ax map-pb), and the matched fragments were filtered using SAMtools ‘view’ (v1.10, -F 256) [34], and ‘falconc bam-filter-clipped’ to remove chimeric match clips (-t -f 0x104). Ratio information was filtered for error correction using a special branch of Racon (v1.6.0; - L - u; https://github.com/isovic/racon/tree/liftover). Lastly, we utilized MUMmer (v4.0.0; https://github.com/mummer4/mummer) to compare our assembled JZ and SZ genomes with the previously released JZV1 and SJV1 genomes to determine the chromosome count and orientation in our assembly.

### Telomere detection and centromere localization

The telomere identification tool (tidk: https://github.com/tolkit/telomeric-identifier) was used to search for the normalized unified sequence TTTAGGG/CCCTAAA within 50 kb of each terminal chromosome sequence. We performed centromere prediction using the quarTeT online analysis platform [35]. Centromics software (https://github.com/ShuaiNIEgithub/Centromics) was used to visualize the repeated distribution of Hi-C and chromatin contact profiles to examine potential centromeres.

### Validation of genome assembly

The completeness of genome assembly was assessed using BUSCO (v4.1.4; parameter - evalue 1e-05). The continuity of genomic assembly was assessed by the location and number of gaps in the genome. To assess genomic consistency, clean reads obtained from second-generation high-throughput sequencing were mapped to the reference genome using BWA (v0.7.17-r1188) software. The continuity of genomic assembly was assessed by the location and number of gaps in the genome. To evaluate the quality and accuracy of genome assembly, *k*-mer statistical results of second-generation data and genome *k*-mer statistical results were used to compare the frequency and distribution of *k*-mer species.

### Gene prediction and functional annotation

The TEs and tandem repeats were annotated via the following workflows. First, we built a *de novo* model sequence based on the reference genome sequence by the use of RepeatModeler (v1.0.11), and used LTR_FINDER and LTR_retriever (v2.9.0) to generate a high­-quality non­-redundant LTR library, and a *de novo* repeat sequence library was built by merging the two *de novo* sequences. Then, the RepBase library and the *de novo* repeat sequence library were merged, and RepeatMasker (v4.0.9) and RepeatProteinMask (v4.0.9) were used to compare and predict repeats and TE_protein type repeats. Finally, all the repeated prediction results were combined to remove redundancy, and the final genome repeat set of combined TEs was obtained.

Gene structure prediction and annotation were combined with transcriptome prediction, homology prediction, and *de novo* prediction. For transcriptome prediction, Illumina clean reads and Oxford Nanopore cDNA clean reads were aligned against the reference genome using Hisat2 (v2.1.0) and Minimap2 (v2.17-r941) [36]. StringTie (v2.1.4) was used to reconstruct the transcript, and TransDecoder (v5.1.0) was used to predict the gene models [37, 38]. For homology-based identification, protein sequences from *Ochetophila trinervis* (Discaria_trinervis_v1), *Rhamnella rubrinervis* (GCA_007844105.2), SJV1 (GCF_020796205.1), JZV1 (LPXJ00000000.2), and DZV1 (GCF_000826755.1) were used for homologous prediction. Tblastn (v2.7.1) and Exonerate (v2.4.0) were used to align the homologous protein sequences to predict transcripts and coding regions based on the alignment results [39, 40]. *De novo* prediction was performed by Augustus (v3.3.2), Genscan (v1.0), and GlimmerHMM (v3.0.4) [41, 42]. Finally, we integrated the gene sets predicted by the three methods into non-redundant and high-confidence gene predictive models with MAKER (v2.31.10) [43]. We employed the BUSCO software (v5.2.2, parameters: -m prot-c 40 -- long-f) to evaluate the quality of genomic annotation.

Functional annotation of the protein-coding genes was implemented by Diamond Blastp (v2.0.11.149; -- evalue 1e-5) searches against the following public databases: UniProt [44], NR [45], GO [46], KEGG [47], and Pathway. InterProScan (v5.52–86.0) and Hmmscan (v3.3.2) softwares were used to obtain conserved sequences, motifs, and domains of proteins by comparison with the databases InterPro and Pfam [48, 49].

### Variation detection between jujube reference genomes

To detect structural variations between genomes, we used Mummer software (v4.0.0; https://github.com/mummer4/mummer) to align JZHapA, JZHapB, DZV2, and SZHapB genomes using the SZHapA genome as a reference. Subsequently, we filtered one-to-one alignments with a minimum alignment length of 100 bp using the delta-filter (delta-filter -i 95 -l 10 000). Next, we employed syri (v1.6; https://github.com/schneebergerlab/syri) to identify collinear regions, structural rearrangements (inversions, translocations, and duplications), and local variants (SNPs, indels). Finally, we utilized Plotsr software [50] for the visualization of structural variants. Coverage observations of HiFi and ONT raw reads at SV sites were performed in IGV.

### Population genetic analyses

We collected leaf samples of 62 cultivars of jujube accessions at the National Jujube Germplasm Repository of China (Laoling, Shandong, China), and 39 wild jujubes were collected from the Jujube Experimental Station of Northwest A&F University (Qingjian, Shaanxi Province, China) and the germplasm repository of Shandong Institute of Pomology (Tai’an, Shandong Province, China) in 2022. Genomic DNA was extracted from leaves, and 150-bp paired-end libraries were constructed for each sample and then sequenced on an Illumina NovaSeq 6000 PE150 as described above. In addition, 571 genome resequencing data were retrieved from NCBI (accession numbers PRJNA560664 and PRJNA306374). Detailed information on the 101 *Z. jujuba* Mill. accessions used for resequencing and the retrieved 571 accessions are provided in Supplementary Data Table S22. The resequencing of each accession was mapped to the JZ genome using BWA-MEM (v0.7.15, 79), and sequence alignment files were then sorted and indexed using SAMtools (v1.3.1) [51]. SNPs were identified using GATK (v1.7.0) and the quality of variation sites was filtered to obtain high-confidence variant sites. According to the QD < 2.0 || MQ < 40.0 || FS > 60.0 || SOR > 3.0 || MQRankSum < -12.5 || ReadPosRankSum < -8’ standard we obtained 30 517 287 high-quality SNPs for subsequent analysis. Using metrics of minor allele frequency (MAF) >0.01 and missing calls >0.9 to filter out low-quality SNPs, we obtained a subset of 522 825 high-confidence SNPs. Then PLINK (v1.90b3.38) [52] was used to filter again (--indep-pairwise 50 10 0.2) and 33 700 SNPs were obtained for phylogenetic, PCA, and population structure analyses. Genome-wide SNPs were used to build NJ phylogenetic trees using the TASSEL software [53]. The phylogenetic tree was colored using the tool iTOL (http://itol.embl.de). PCA was performed with Tassel software and the first two eigenvectors were plotted. Population structure was analyzed using ADMIXTURE (v1.3) [54]. To explore the best genetic cluster subpopulations (*K*), we predefined the number of genetic clusters *K* from 2 to 6 and ran the cross-validation error (CV) procedure. LD was calculated using PLINK software (www.cog-genomics.org/plink2). LD coefficients (*r*^2^) of all chromosomes were analyzed according to a 1000-kb window.

### Genome scanning for divergent regions

To analyze the selection signal of the whole genome, nucleotide diversity (π) and population fixation statistics (*F*_ST_) were calculated for 289 wild jujubes and 359 cultivated jujubes using VCFtools software, with a sliding window size of 100 kb and a sliding step of 10 kb. Perl scripts were used to calculate inter-group ROD values based on the formula ROD = 1 − (Pi pop1)/(Pi pop2). The values of ROD and *F*_ST_ were both in the top 5% and were selected as the selective signal.

### Transcriptome analysis and homologous gene identification

We aligned the RNA-seq clean reads (PRJNA306374) from three developmental stages of JZ and SZ fruits with the respective genome sequences [4]. FeatureCounts was used for counting and the R package GenomicFeatures was used to standardize expression, which was visualized using the R library. According to Zhou *et al*. [55] workflow, the alleles of JZ and SZ haplotype genomes were identified using MCScanX and BlastP software (v2.5.0). *K*_a_ and *K*_s_ were calculated in TBtools [ 56] using coding and protein sequences.

## Supplementary Material

Web_Material_uhae071

## Data Availability

The raw sequence data of whole-genome sequencing and transcriptome sequencing in *Z. jujuba* Mill. and *Z. jujuba* var. *spinosa*, as well as the assemblies, have been deposited in NCBI project PRJNA974227 (https://www.ncbi.nlm.nih.gov/bioproject/PRJNA974227/). The whole-genome resequencing data were deposited in CNGB project PRJCA015614 (https://ngdc.cncb.ac.cn/gsa/s/VZxLq3Z8). The annotation files for protein-coding genes, along with other related files for genome assembly, have been stored in figshare, an online data repository, at https://figshare.com/s/ad5d747ccc2ccbb2b65b.
