# Supplementary material for: Haplotype-resolved T2T reference genomes for wild and domesticated accessions shed new insights into the domestication of jujube
Source: Hortic Res. 2024 Mar 7;11(5):uhae071. doi: 10.1093/hr/uhae071 (PMC11079485; doi:10.1093/hr/uhae071)
Supplement: Web_Material_uhae071 [file web_material_uhae071.zip › Supplementary Figures.docx]

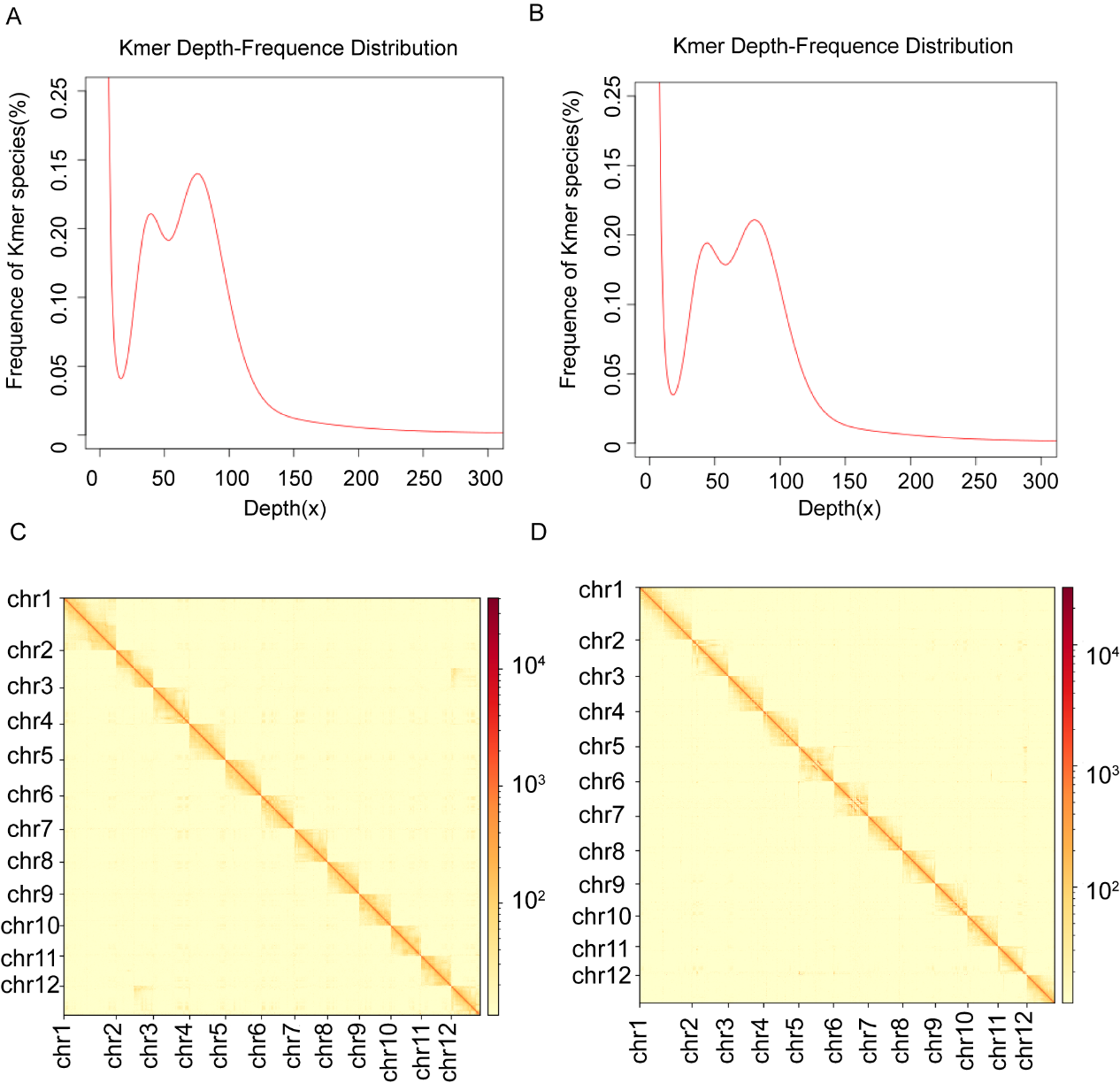


**Figure S1 Genome size and heterozygosity assessment**

(A), (B) Kmer-depth and Kmerspecies-Frequence distribution of JZ (A) and SZ(B) at Kmer=19. The horizontal coordinate means the depth at Kmer=19, and the vertical coordinate means the frequency at different depths.


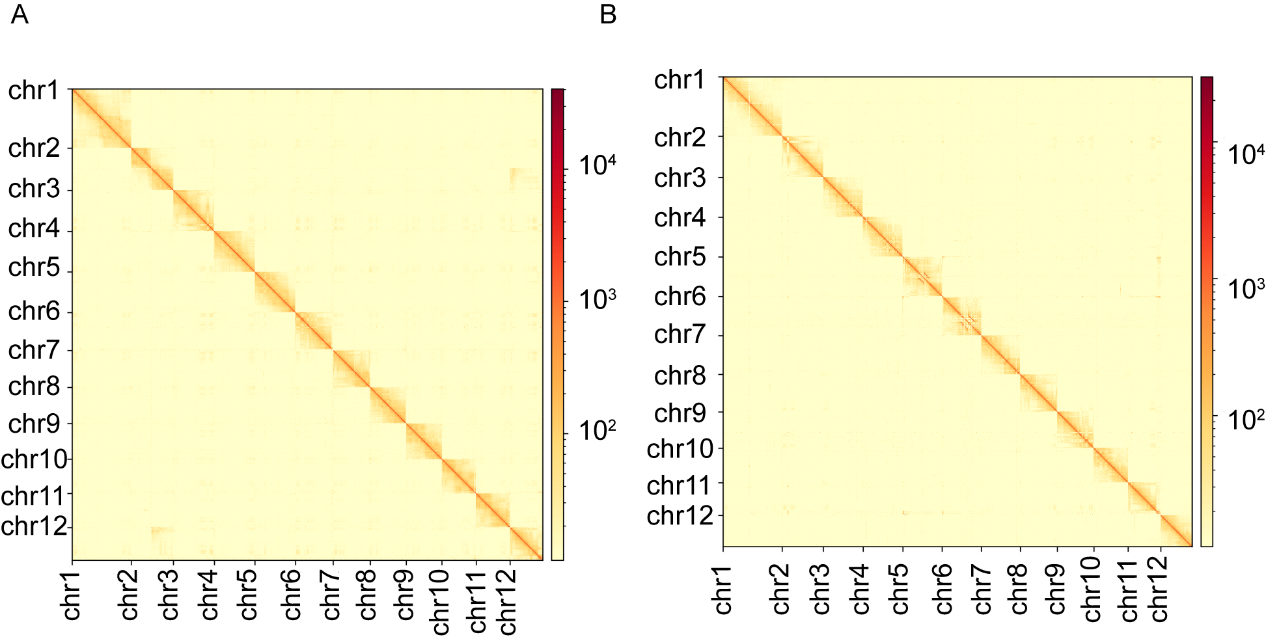


**Figure S2 Preliminary assembly assembly of Hi-C links interactive heat map.**

(A), (B) Chromosome HIC interaction heat map of JZ(A) and SZ(B). The binary logarithm of each link number is coded using colors ranging from light yellow to dark red, indicating the frequency of Hi-C interaction links from low to high.

**Figure S3 Heatmap of chromosome interactions in JZ and SZ final assembly genome.**
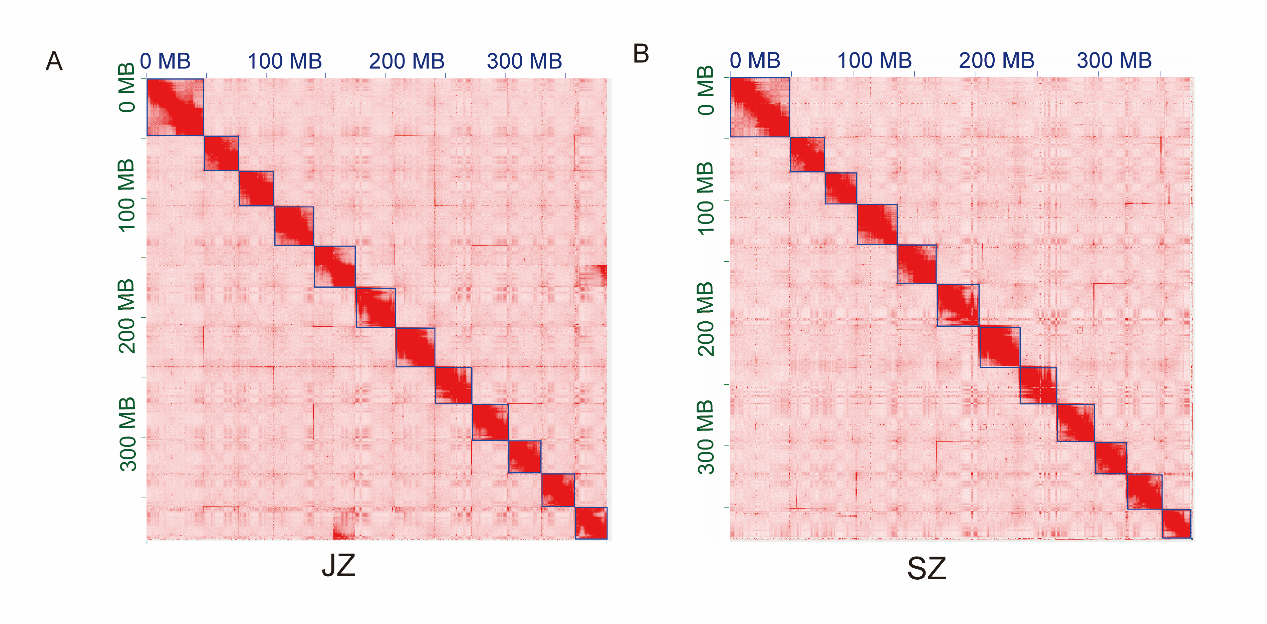


Horizontal and vertical coordinates indicate the position of chromosomes. Blue box lines represent each chromosome. The darker the red color, the higher the contig interaction intensity.


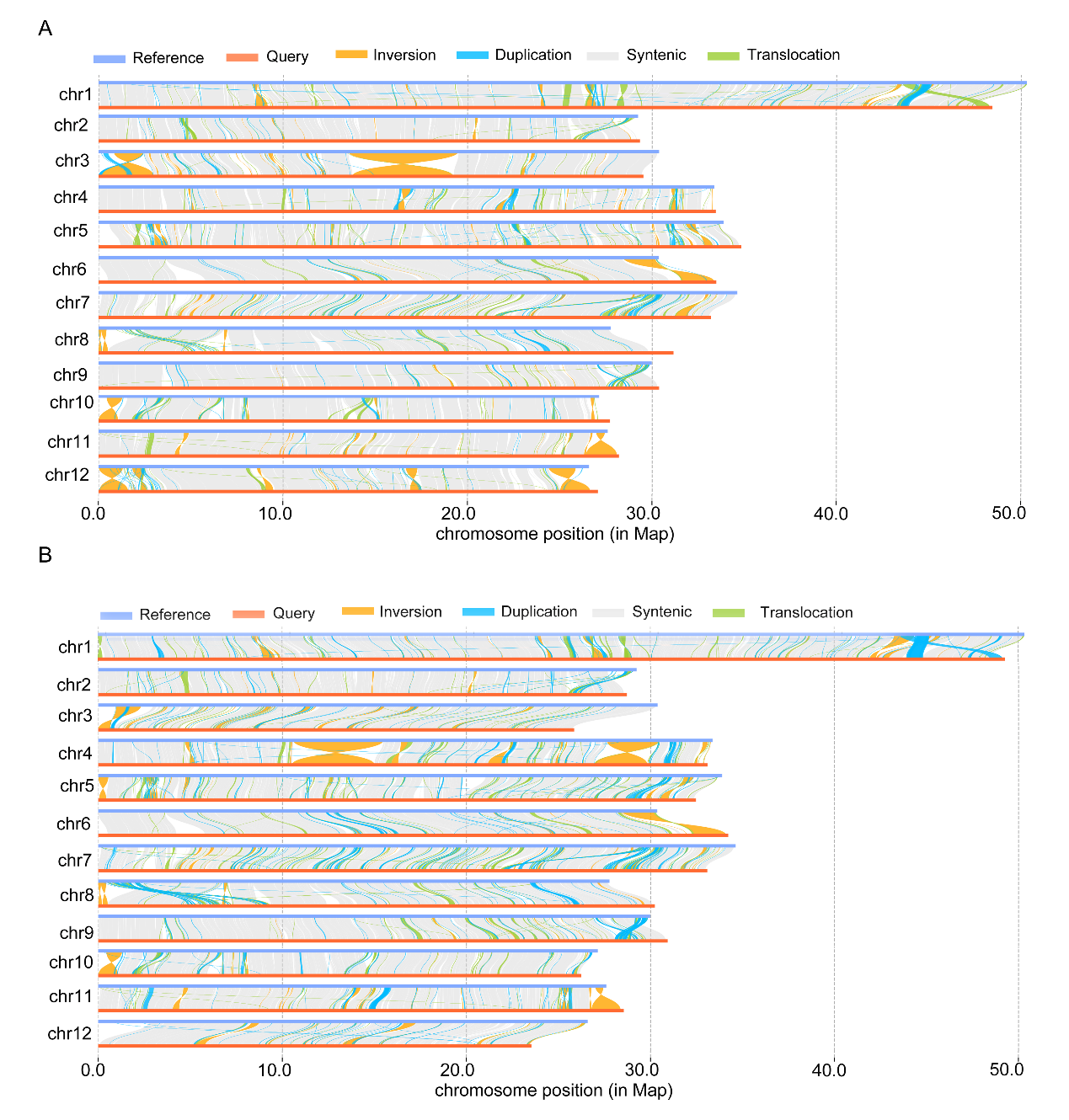


**Figure S4** **Syntenic analysis of the primarily assembled JZ and SZ genomes with published JZV1 and SJV1 genomes.**

1. Syntenic analysis of the primary assembled JZ genome with the published JZV1 genome.
2. Syntenic analysis of the primary assembled SZ genome with the published SJV1 genome.

The query chromosome represents the newly assembled version, and reference represents the published version. Syntenic areas and SV variations are highlighted in different colors, gray for collinear areas, yellow for inversion, cyan for translocation, and blue for repetition. The horizontal coordinate indicates chromosome length.

**Figure S5
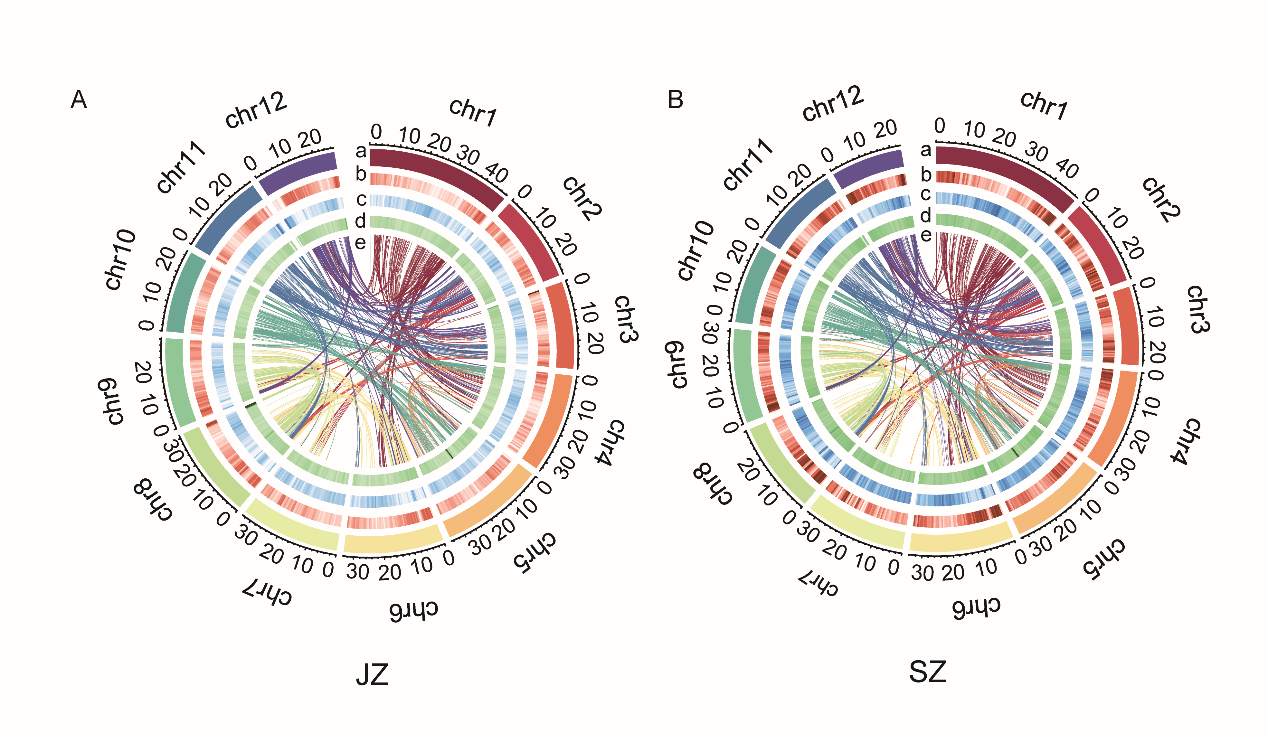
 Genomic characteristics of 12 chromosomes of JZ and SZ chimera genomes**.

(a) Chromosome karyotype, (b) gene density, (c) repeat sequence density, (d) GC content, and (e) collinearity between chromosomes. Central colored lines represent syntenic blocks; block size = 50 kb.


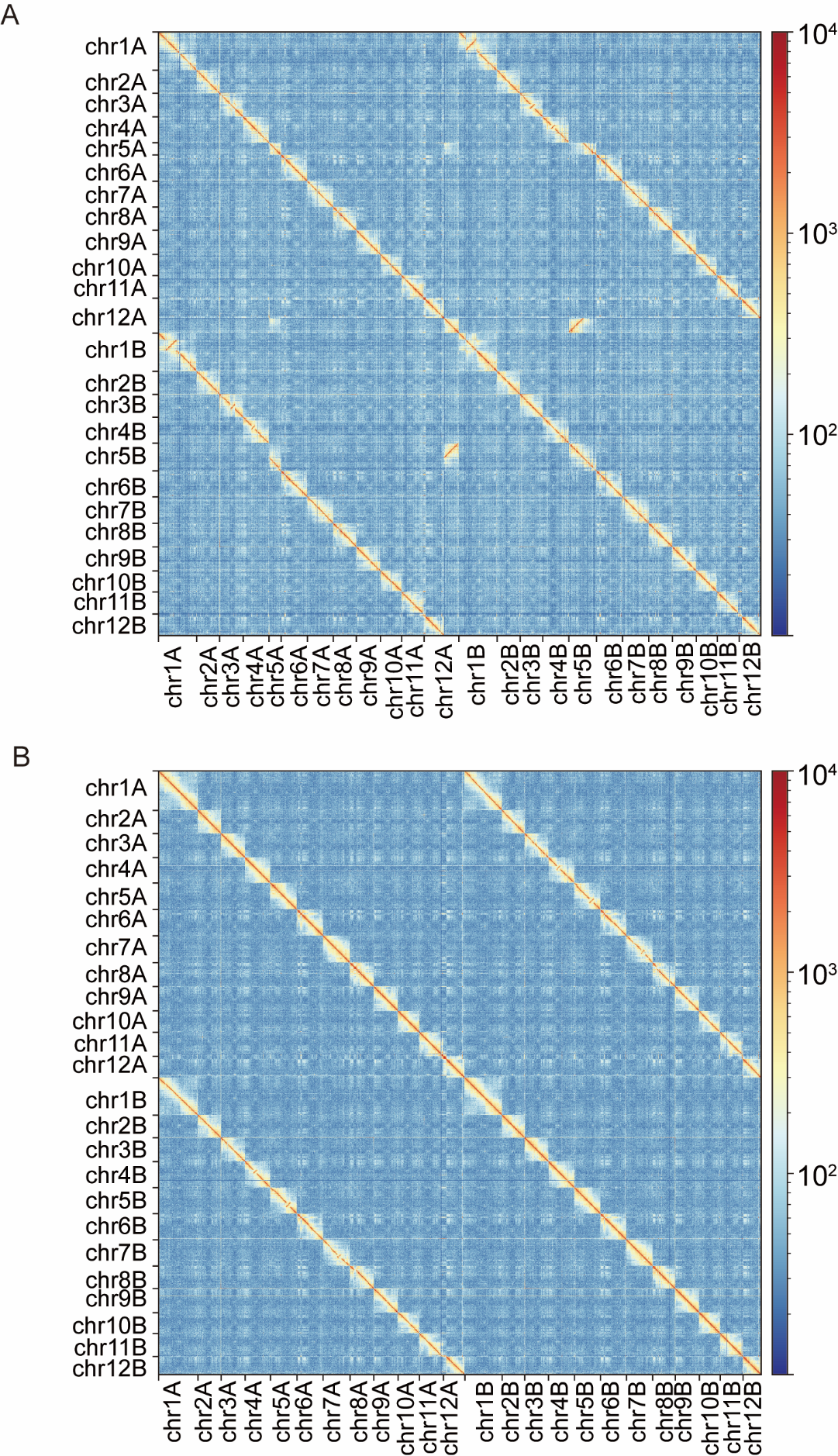


**Figure S6** **Haplotype assembly HIC anchoring heat maps of JZ(A) and SZ(B).**


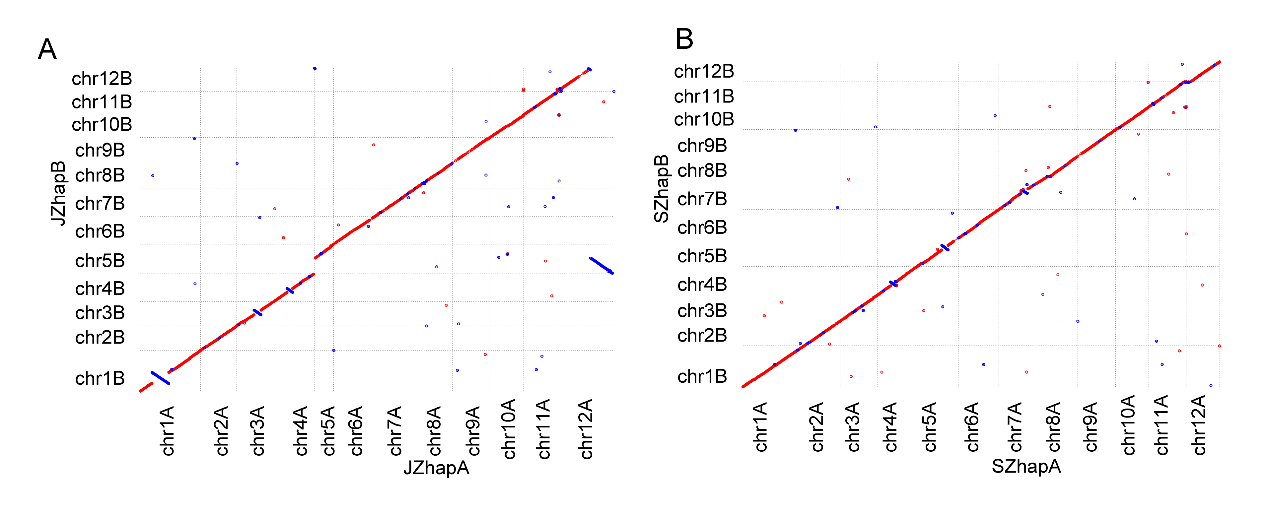


**Figure** **S7 Haplotype-resolved genomes syntenic analysis of JZ and SZ.**

(A) Two haplotype genomic collinearity maps of JZ. (B) Two haplotype genomic collinearity maps of SZ.


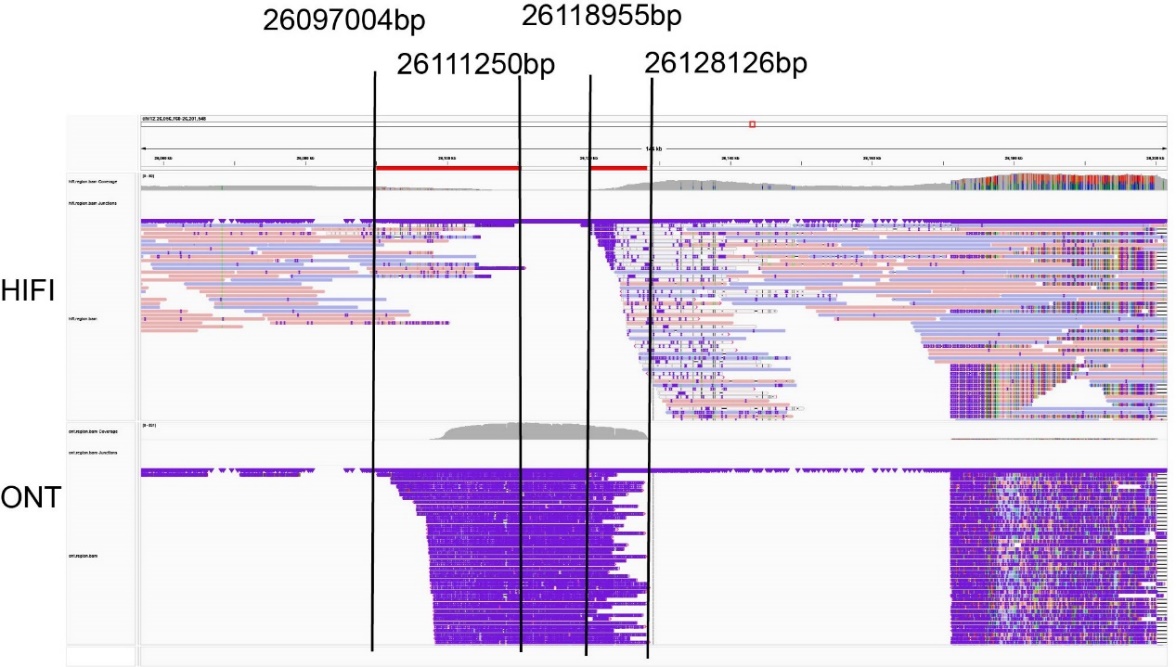


**Figure S8 The chromosome coverage of JZHapA12 (26051435bp-26196224bp) was observed by HIFI and ONT read in IGV.**

**Figure S9 HIC interaction map of chr5 and chr12 chromosomes of JZ**. **
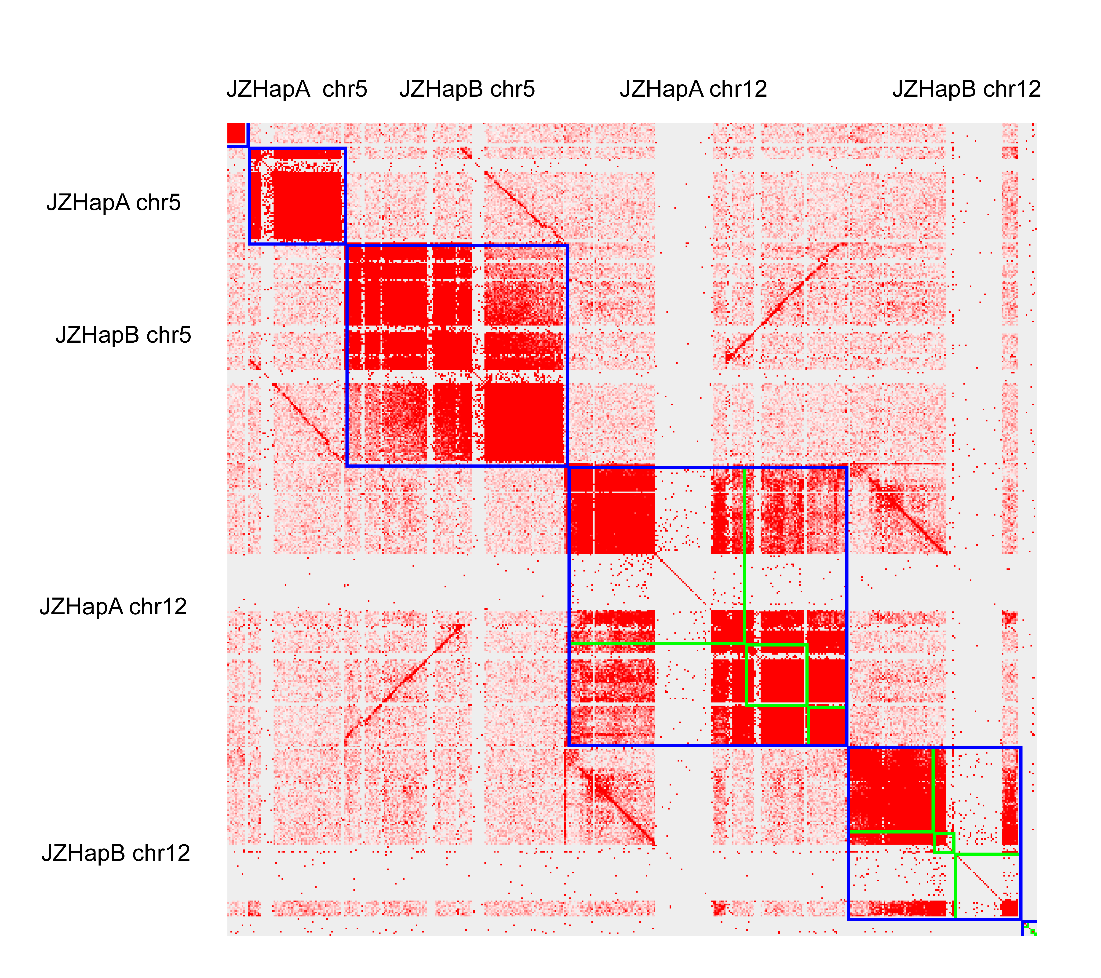
**

**
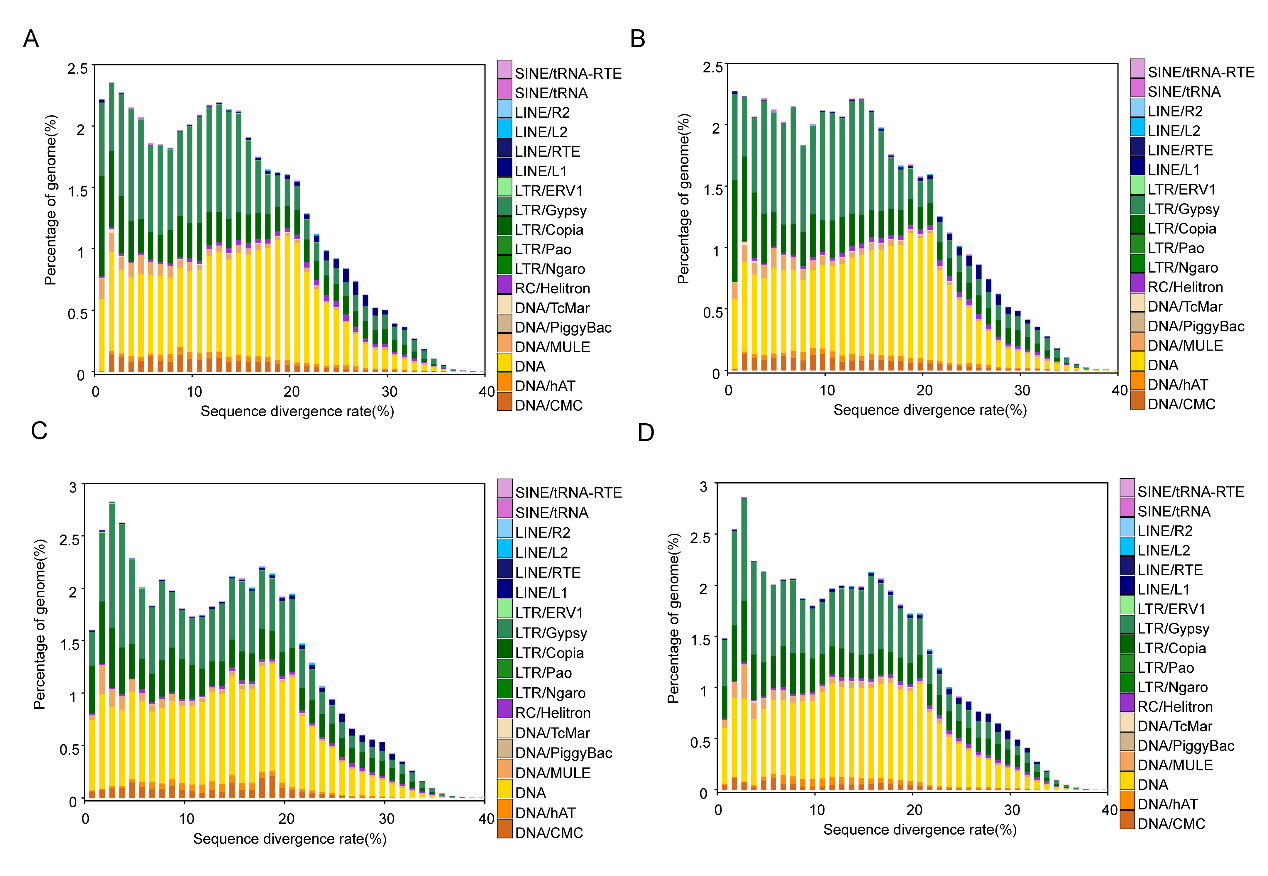
**

**Figure S10** **Distribution of sequence divergence rates of JZ and SZ genome.**

(A) Distribution of sequence divergence rates of JZHapA.

(B) Distribution of sequence divergence rates of JZHapB.

(C) Distribution of sequence divergence rates of SZHapA.

(D) Distribution of sequence divergence rates of SZHapB.


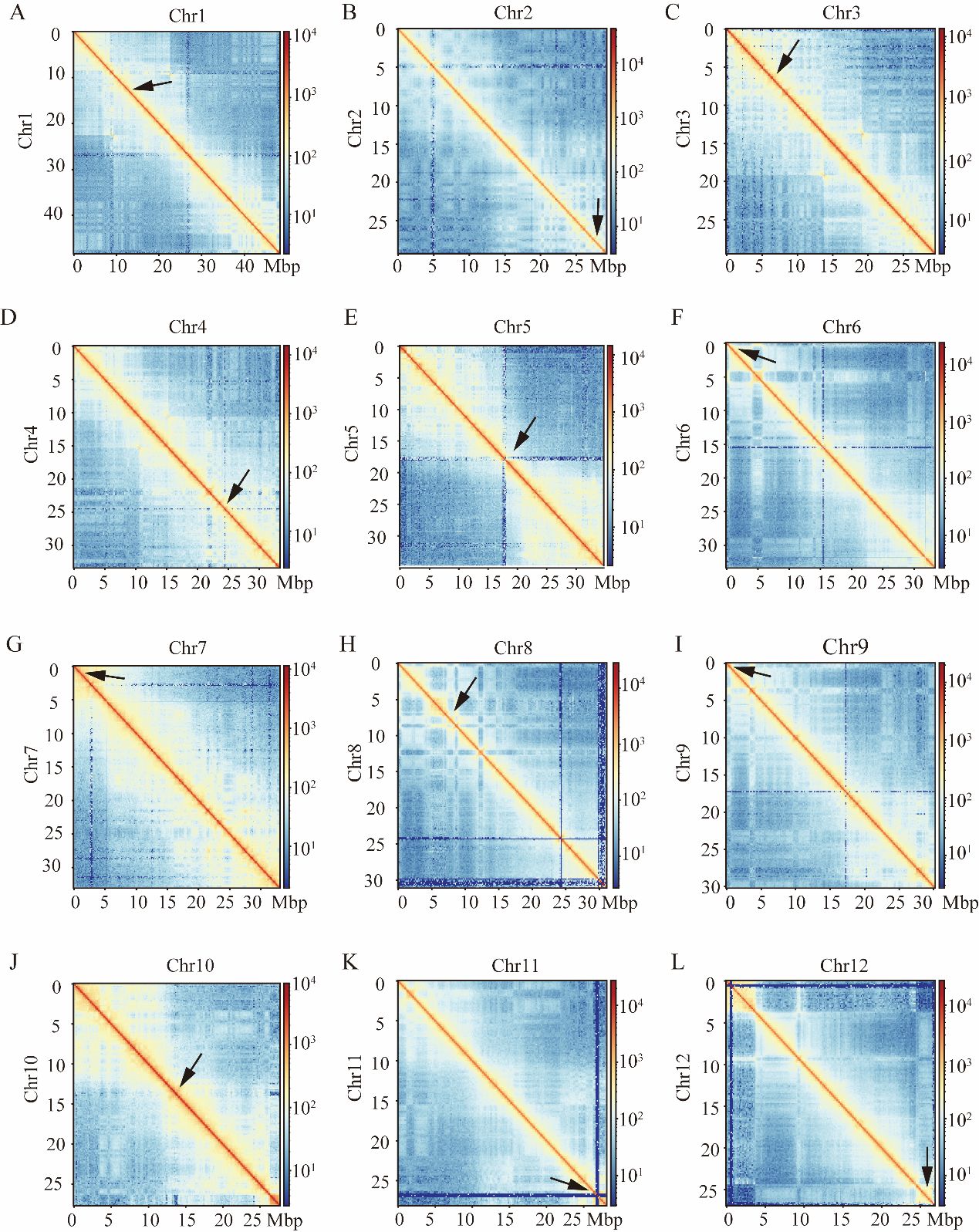


**Figure S11** **Visualization of the potential centromere region of the JZ genome.**

Arrows indicate potential centromere regions.


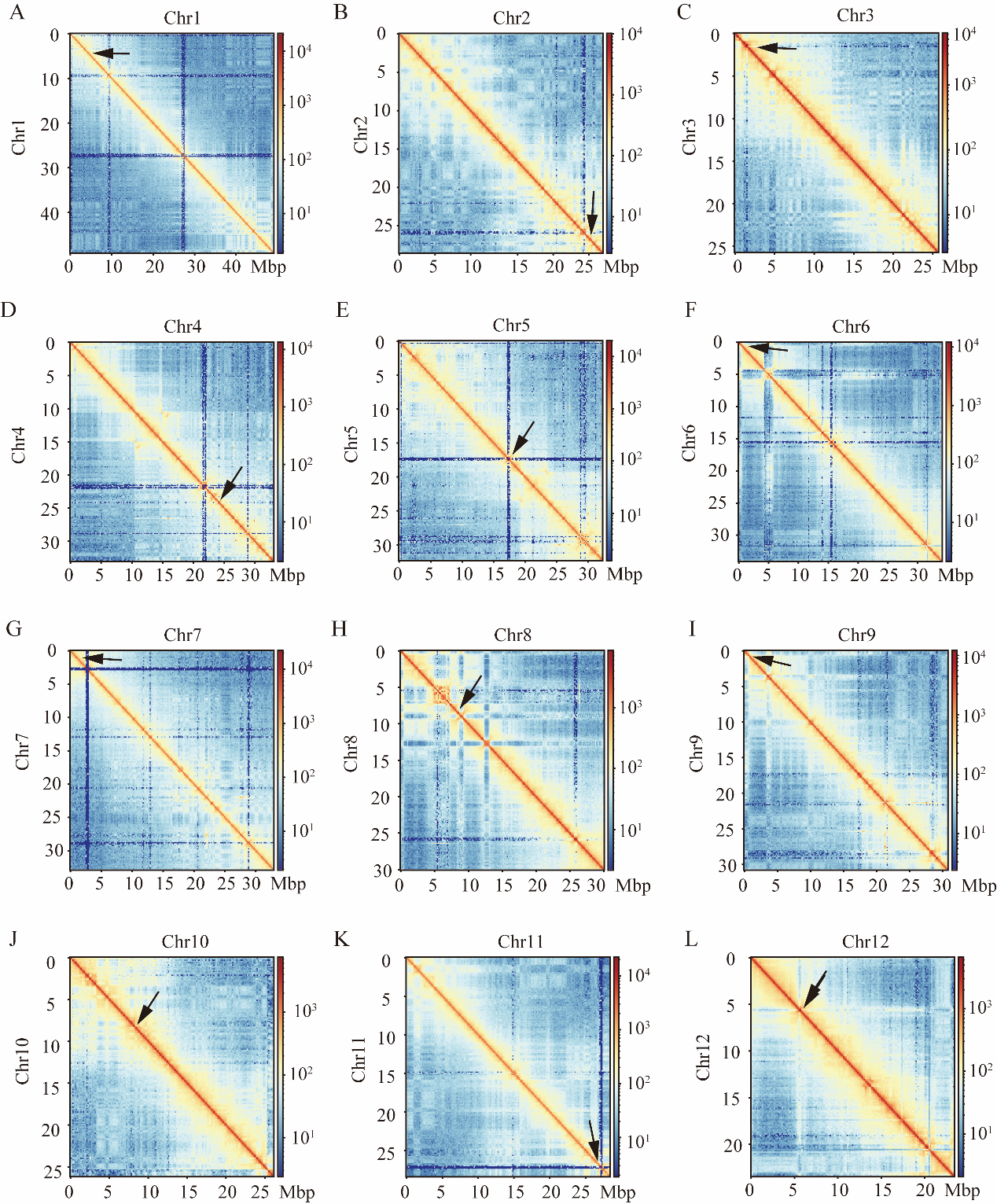


**Figure S12** **Visualization of the potential centromere region of the SZ genome.**

Arrows indicate potential centromere regions.


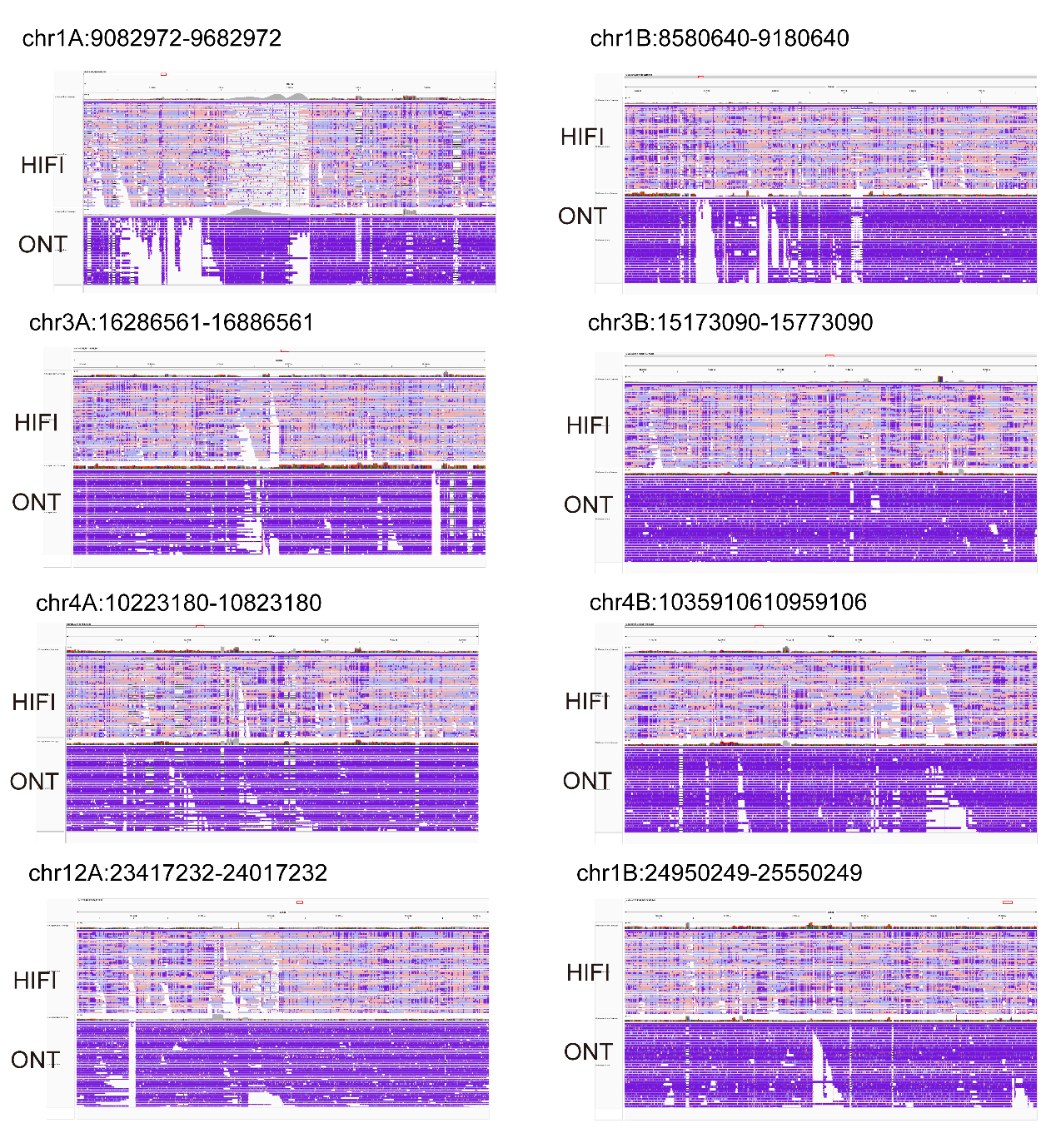


**Figure S13** **Observation of HIFI and ONT reads coverage of structure variation sites in JZ haplotype genome.**


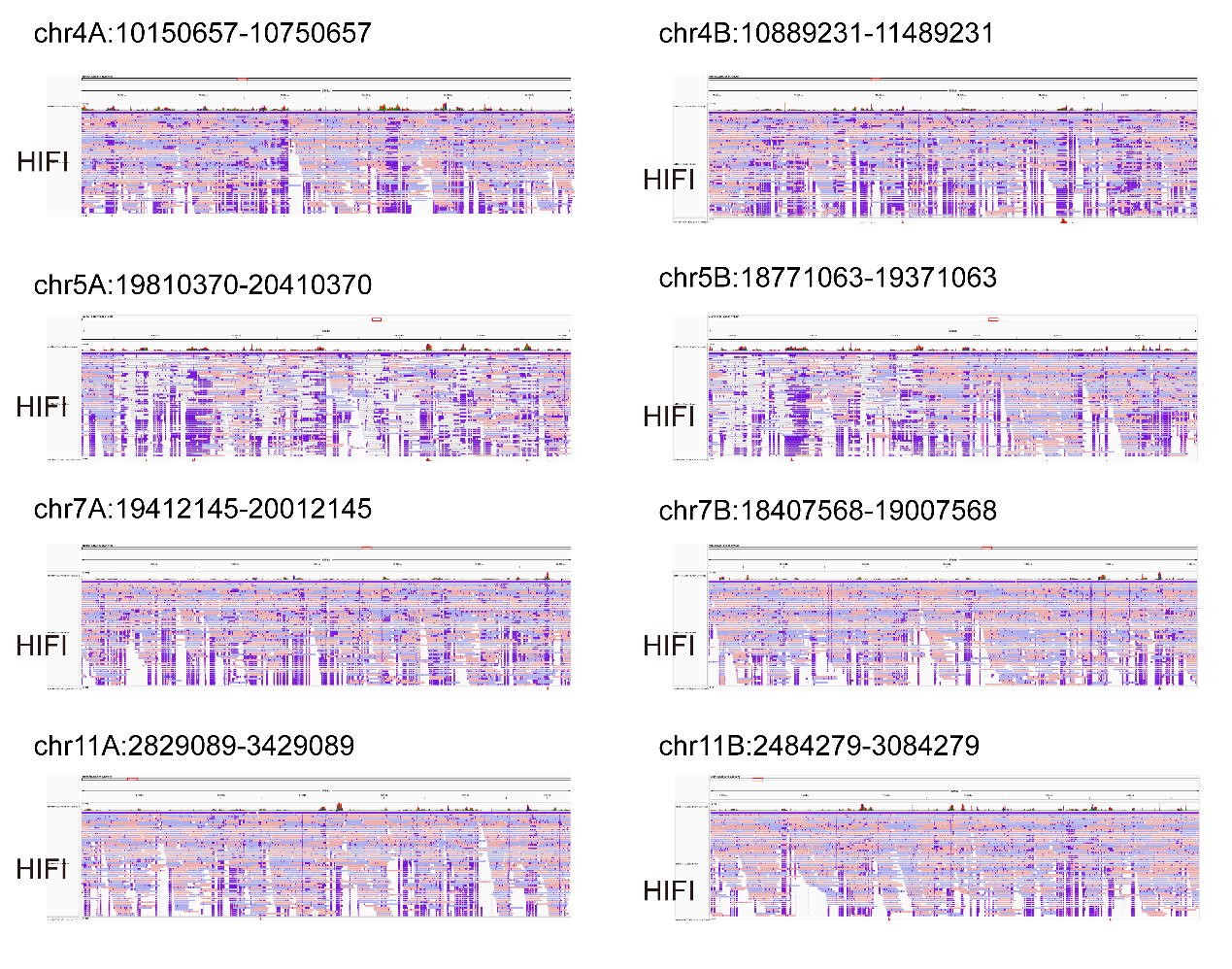


**Figure S14 Observation of HIFI reads coverage of structure variation sites in SZ haplotype genome.**


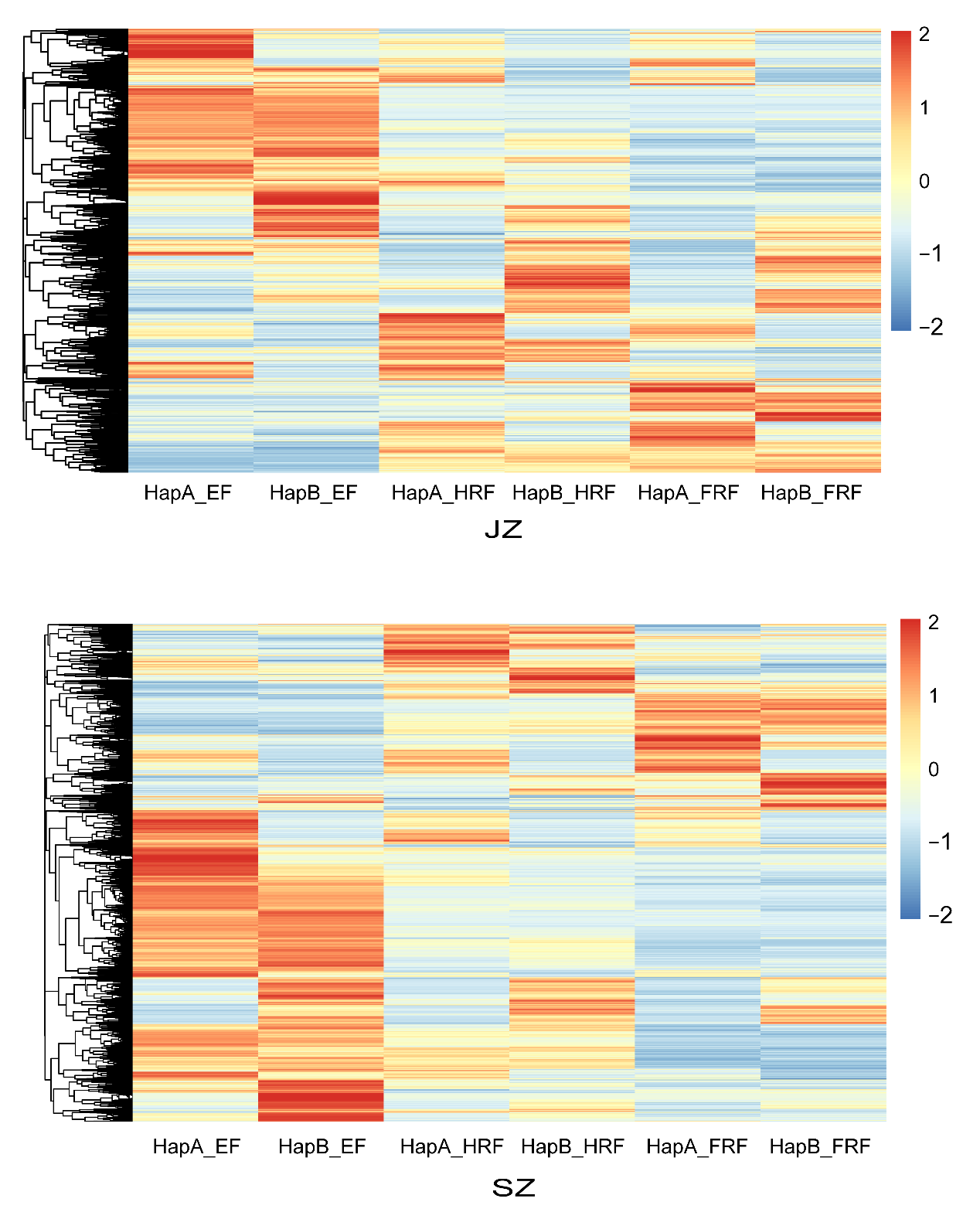


**Figure S15. Expression of alleles in the two haplotype genomes of JZ and SZ in fruits at different developmental stages.**

Note: EF, expanding fruit; HRF, half-red fruit; and FRF, full-red fruit.

**
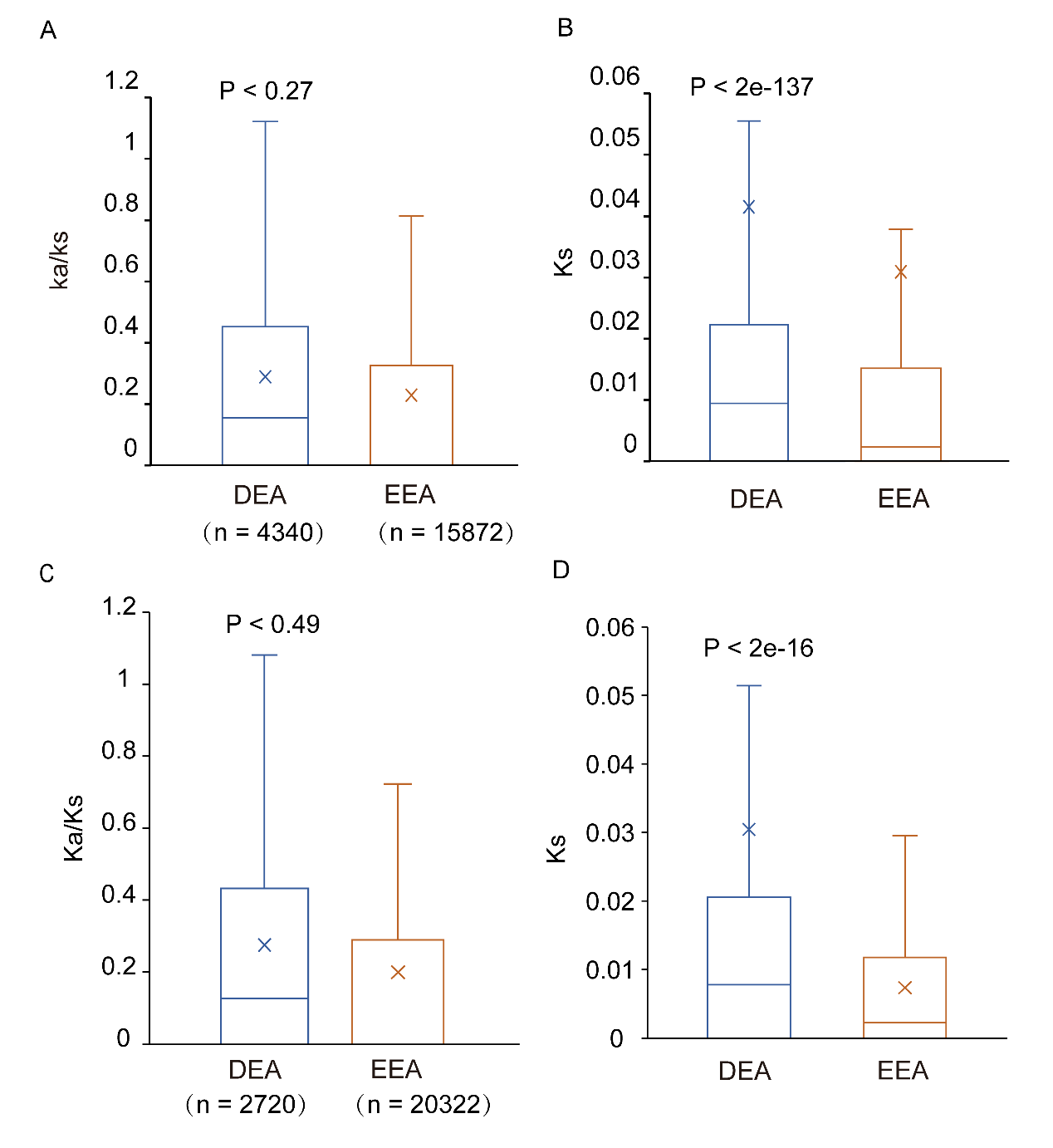
**

**Figure S16 Comparison of Ka/Ks ratio and Ks value between DEA and EEA genes.**

(A) Comparison of Ka/Ks ratio in JZ haplotype genome. (B) Comparison of Ks ratio in JZ haplotype genome. (C) Comparison of Ka/Ks ratio in SZ haplotype genome. (D) Comparison of Ks ratio in SZ haplotype genome. The p-value was calculated using the Wilcoxon test.

**Figure S17 KEGG pathway enrichment analysis of DEA genes in JZ and SZ
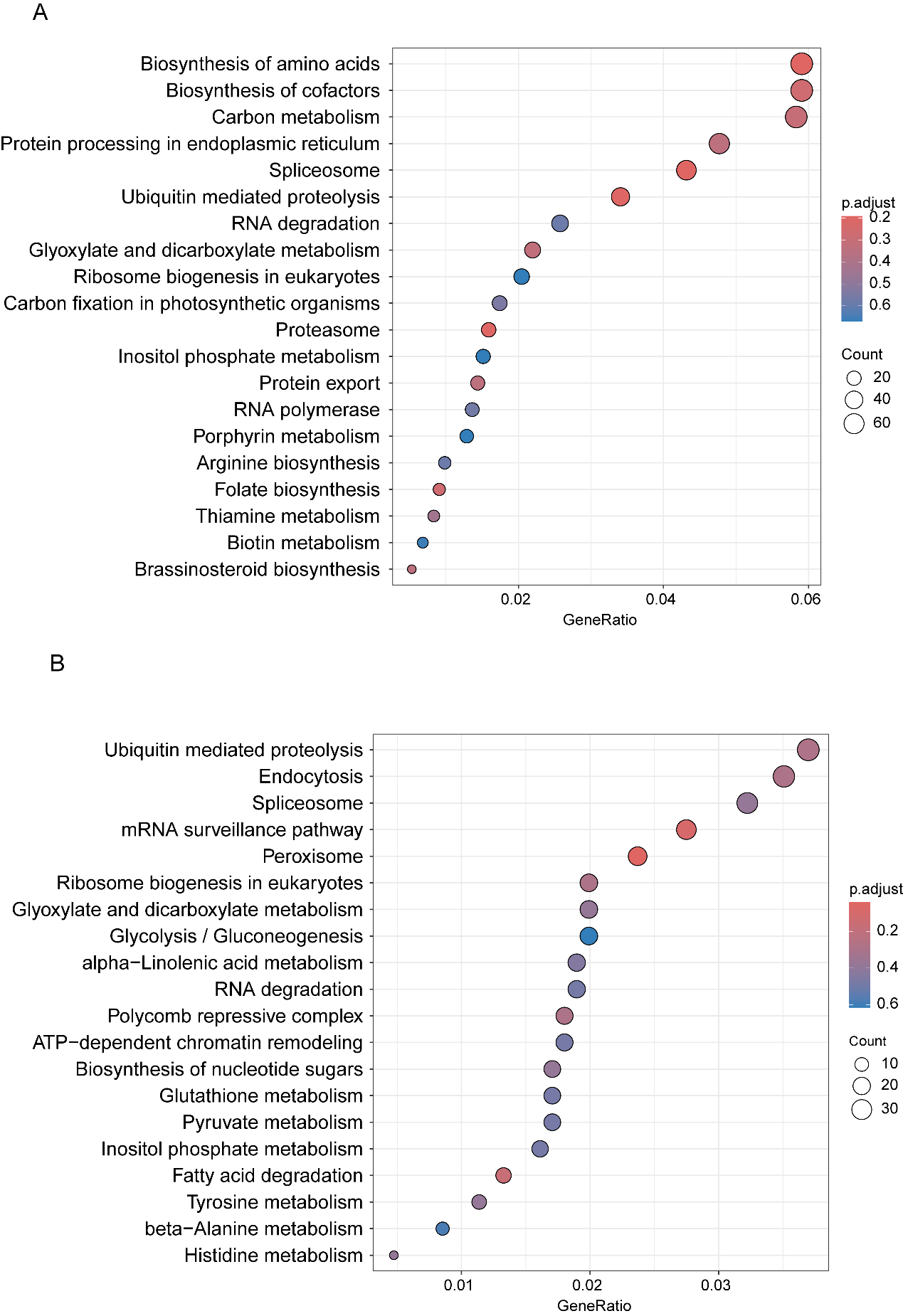
**


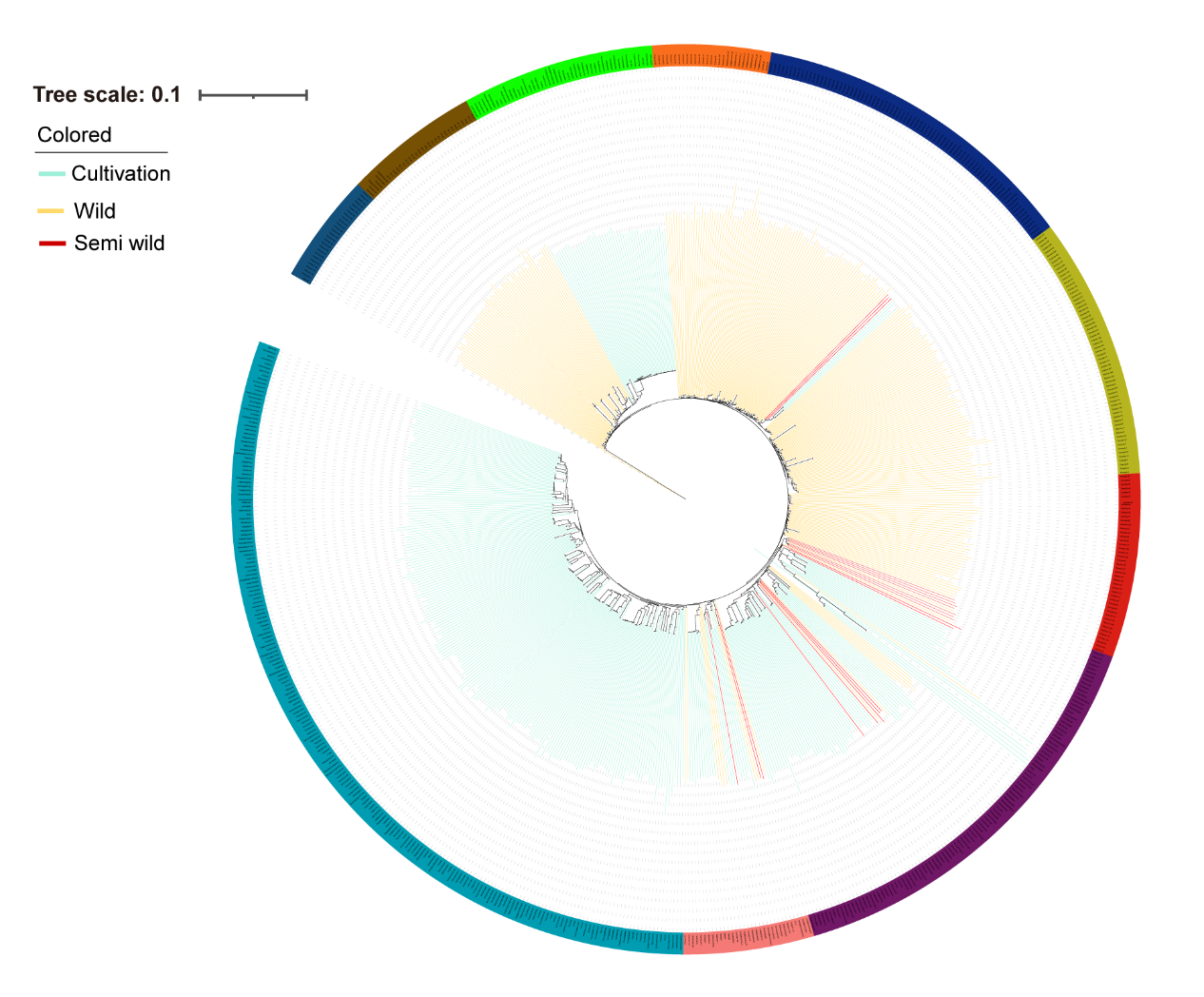


**Figure S18 Neighbor-joining phylogenetic (NJ) tree of jujube and wild jujube accession inferred from the whole-genome SNPs.**


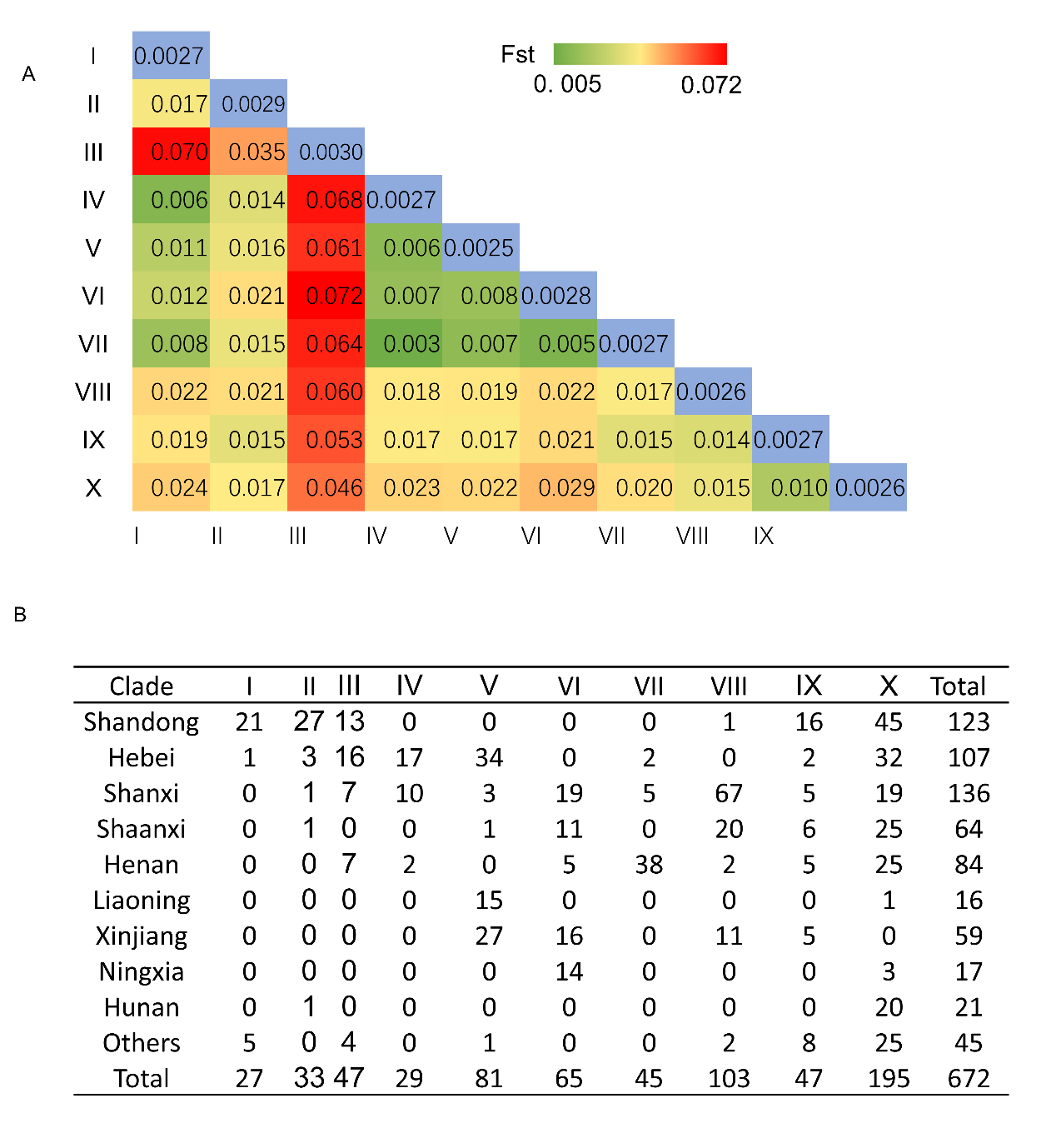


**Figure S19** **Population distribution and population differentiation.**

(A) Population differentiation in each clade (subgroup) of the phylogenetic tree. The values in the blue diagonal cells report nucleotide diversity (π) within each group.

(B) The geographic origin of each accession in the ten clades.


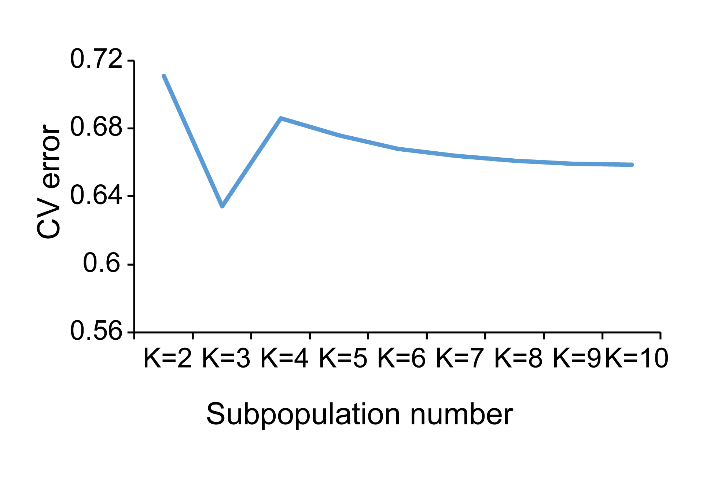


**Figure S20 Visualizing CV errors and determining the ideal cluster number.**


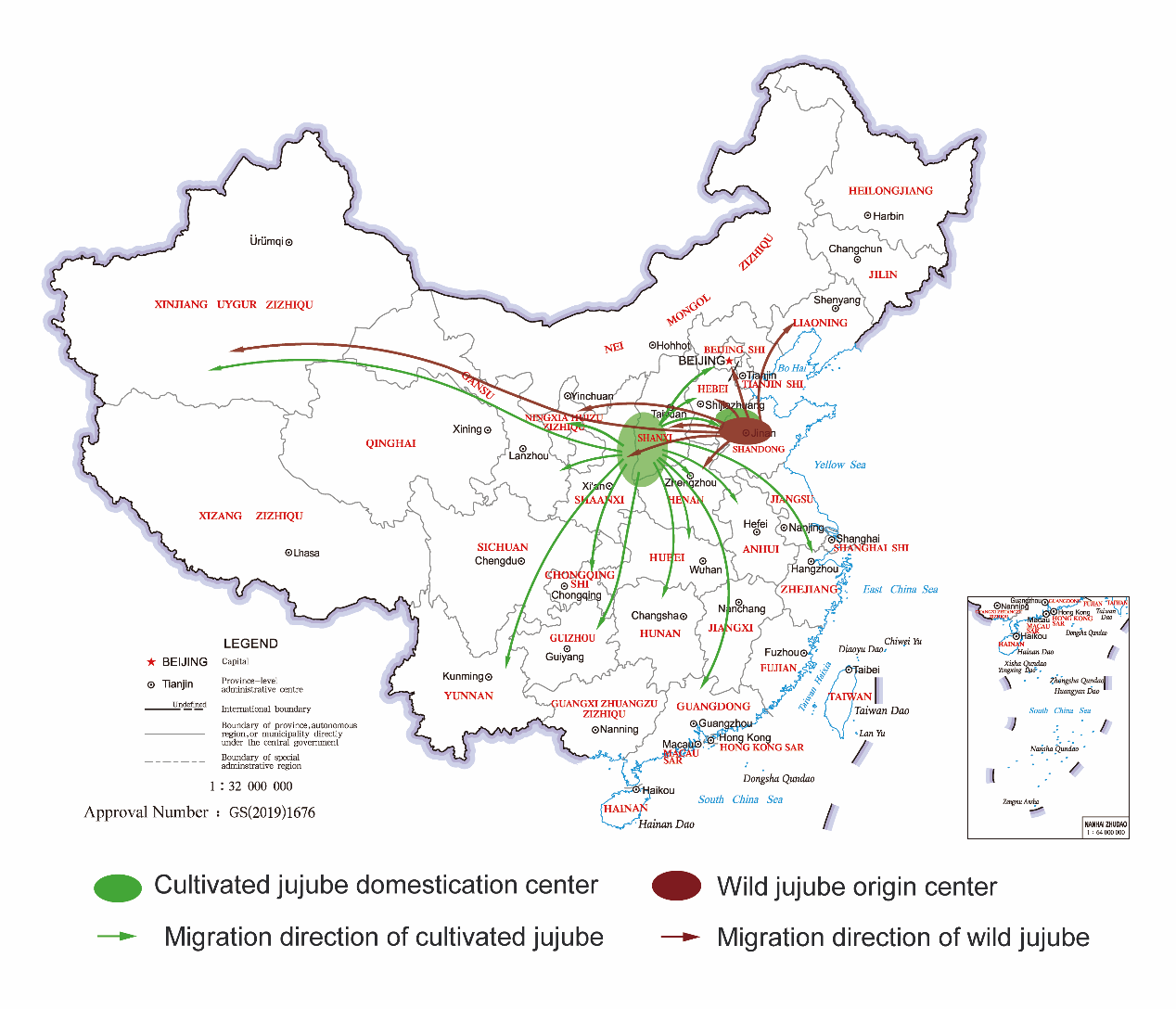


**Figure S21 Deduced of migration routes of wild jujube and cultivated jujube from centers of origin and domestication to other regions.**

The green and purple ellipses represent the origin center of wild jujube and the domestication center of cultivated jujube. The purple and green arrows represent the migration direction of wild and cultivated dates respectively. The map is based on the standard map No. GS (2019)1676 of the standard map service website (http://bzdt.ch.mnr.gov.cn/index.html), and without changes to map boundaries.


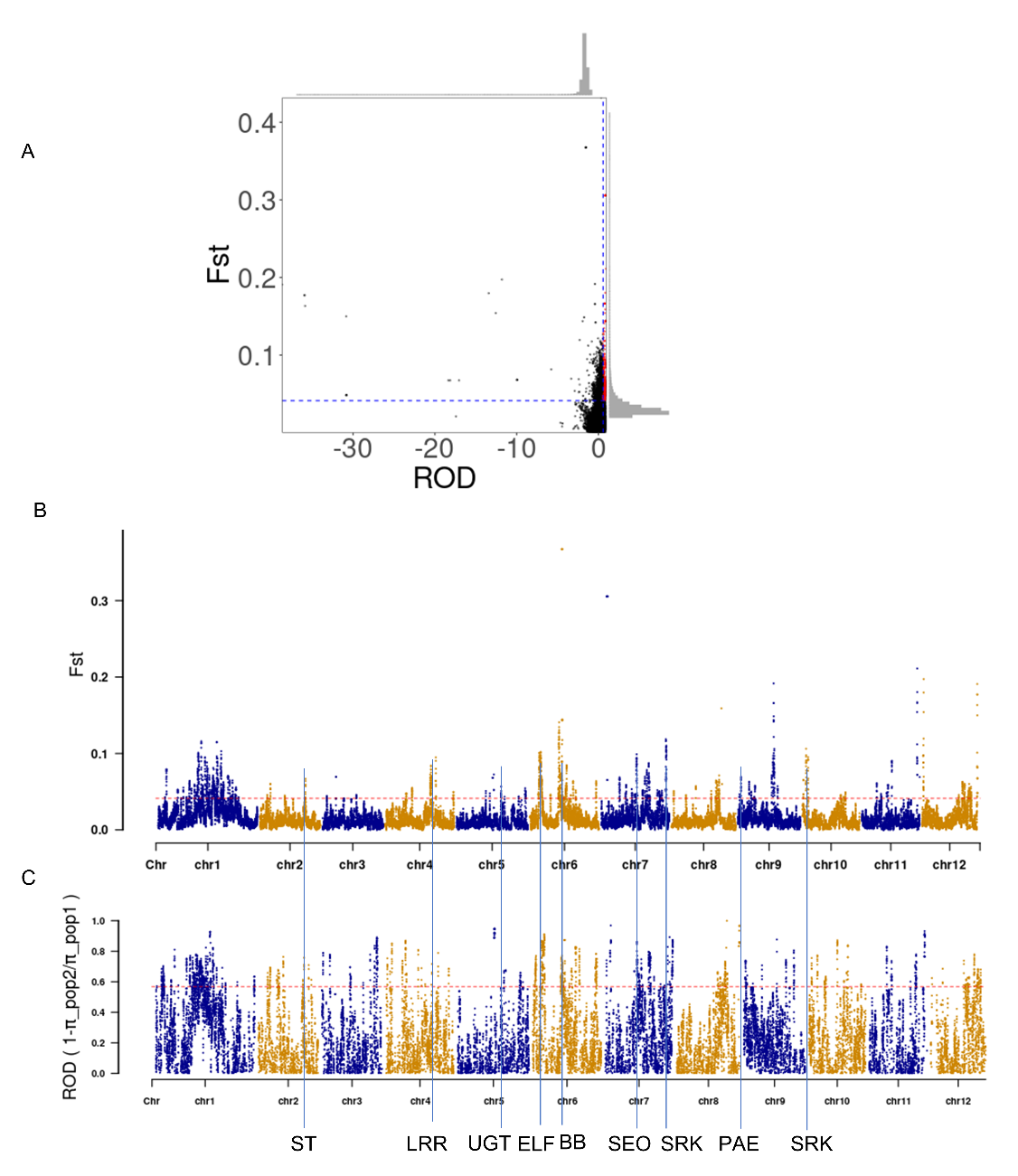


**Figure S22** **Genome-wide screen of selective sweep regions between wild and cultivated accession.**

(A) Genome-wide screen of selected regions based on ROD and population differentiation (FST). The red area is the selected regions shared by both Fst and ROD, which were identified as selected regions for two groups.

(B) The Manhattan map shows the selective sweeps region of the jujube genome. Regions with both FST values and p ratios in the top 5% were regarded as having domestication, with a 100 kb window sliding in 10 kb steps signals.  Candidate genes in the selective regions are indicated by black arrows.

(C) ROD values among the whole genome between wild and cultivated accession. Red horizontal dashed lines indicate the genome-wide threshold of selection signals and population differentiation index. Pop1means wild accession, pop2 means cultivation accession. UGT, UDP-glycosyltransferase 89B2-like；SEO, Sieve element occlusion N-terminus; SRK, G-type lectin S-receptor-like serine; BB，BIG BROTHER; LRR, Leucine rich repeat; PAE, Pectinacetylesterase; ELF, Early flowing.
